# Transcriptional Regulation of Metabolic and Cellular Processes in Durum Wheat (*Triticum turgidum* subsp. *durum*) in the Face of Temperature Increasing

**DOI:** 10.3390/plants10122792

**Published:** 2021-12-16

**Authors:** Luis Abraham Chaparro-Encinas, Gustavo Santoyo, Juan José Peña-Cabriales, Luciano Castro-Espinoza, Fannie Isela Parra-Cota, Sergio de los Santos-Villalobos

**Affiliations:** 1 Instituto Tecnológico de Sonora, 5 de Febrero 818 Sur, Ciudad Obregón 85000, Sonora, Mexico; luis.chaparro15278@potros.itson.edu.mx (L.A.C.-E.); lcastro@itson.edu.mx (L.C.-E.); 2 Departamento de Fitomejoramiento, Universidad Autónoma Agraria Antonio Narro (UAAAN) Unidad Laguna, Periférico Raúl López Sánchez, Valle Verde, Torreón 27054, Coahuila, Mexico; 3 Instituto de Investigaciones Químico Biológicas, Universidad Michoacana de San Nicolás de Hidalgo, Morelia 58000, Michoacán, Mexico; gustavo.santoyo@umich.mx; 4 Centro de Investigación y de Estudios Avanzados, Unidad Irapuato, Libramiento Norte Carretera Irapuato León Kilómetro 9.6, Carr Panamericana Irapuato León, Irapuato 36821, Guanajuato, Mexico; juan.pena@cinvestav.mx; 5 Campo Experimental Norman E. Borlaug, Instituto Nacional de Investigaciones Forestales, Agrícolas y Pecuarias (INIFAP), Norman E. Borlaug Km. 12, Valle del Yaqui, Ciudad Obregón 85000, Sonora, Mexico

**Keywords:** RNA-Seq, ROS, climate change, abiotic stress

## Abstract

The Yaqui Valley, Mexico, has been historically considered as an experimental field for semiarid regions worldwide since temperature is an important constraint affecting durum wheat cultivation. Here, we studied the transcriptional and morphometrical response of durum wheat at an increased temperature (+2 °C) for deciphering molecular mechanisms involved in the thermal adaptation by this crop. The morphometrical assay showed a significant decrease in almost all the evaluated traits (shoot/root length, biovolume index, and dry/shoot weight) except in the dry root weight and the root:shoot ratio. At the transcriptional level, 283 differentially expressed genes (DEGs) were obtained (False Discovery Rate (FDR) ≤ 0.05 and |log2 fold change| ≥ 1.3). From these, functional annotation with MapMan4 and a gene ontology (GO) enrichment analysis with GOSeq were carried out to obtain 27 GO terms significantly enriched (overrepresented FDR ≤ 0.05). Overrepresented and functionally annotated genes belonged to ontologies associated with photosynthetic acclimation, respiration, changes in carbon balance, lipid biosynthesis, the regulation of reactive oxygen species, and the acceleration of physiological progression. These findings are the first insight into the regulation of the mechanism influenced by a temperature increase in durum wheat.

## 1. Introduction

In the Yaqui Valley, the birthplace of the Green Revolution, agriculture is based on intensive practices such as monoculture, mechanization, and large-scale agrochemical applications. This region is responsible for approximately 54% of the national wheat production, mainly durum wheat (*Triticum turgidum* subsp. *durum*) [1]. However, this valley is susceptible to elevated temperatures [2,3], which puts the maintenance of food production at risk [4,5,6]. Climate is one of the most important determinants of durum wheat yield and accounts for 30% to 50% of the overall output variability [7]. Therefore, crop growth and yield under rising temperature scenarios are receiving increased attention. For example, the temperature effect has been calculated to reduce the durum wheat yield from 4.1% to 6.4% per Celsius degree increase [8], and a loss of yield of 24% under a growth temperature of 31 °C in the flowering stage [7].

Plants respond to elevated temperatures in various ways; an increase of 2 °C to 5 °C above the optimal conditions can lead to a process known as thermomorphogenesis, which consists of changes in development and morphology influenced by a moderate increase in temperature [9]. The physiological effects of thermomorphogenesis are characterized by a fully expanded leaf structure, a larger root system, and early flowering. These are aimed at reducing the exposure of meristematic tissue to adverse conditions [10]. A temperature above this threshold of 5 °C to 10 °C, from this point called heat stress, can lead to a decline in pollen viability, starch synthesis, and grain filling. Additionally, severe thermal stress conditions can produce seed sterility due to the sensitivity of microspore and megaspore development [11].

Durum wheat cultivars developed in tropical regions are mostly adapted to short periods of temperatures above 20–30 °C; however, the temperature-influenced growth could have negative implications depending on exposure time, i.e., 5 days at temperatures above that range. Thus, an increase in temperature impacts yield-related traits, such as the thousand-kernel weight and reproductive tiller number, explaining approximately 52% of phenotypic variance [12].

In durum wheat, the stages most sensitive to heat stress are anthesis and the vegetative period; both stages are characterized by being crucial for the acquisition of nutrients (vegetative stage) and the grain filling (anthesis) [11]. Physiologically, the effect of heat stress is presented by a reduced photosynthetic rate, accelerated development, reduced flowering time, and fewer grains per spike [13]. It has been observed that the negative effects on yield may be imperceptible up to an upper limit of 31 °C near the flowering stage. This may depend on the genotype, the availability of water, and the stage of development [14]. A temperature above 31 °C could inhibit the transport of photoassimilates and nitrogen compounds from leaves and stems to grains [10,15].

The application of multi-omics approaches has allowed the identification of several molecular mechanisms of response to heat stress in model plants (*Arabidopsis* spp.), although these mechanisms are diverse and depend on specific conditions. The main temperature sensors in plants have been reported to be phytochrome B (PhyB) and phototropin, which respond to moderate changes in basal temperature and variation in red light. Once warm temperatures and red light are detected, PhyB promotes the accumulation of interactive phytochrome factors (PIF (bHLH family of transcription factors)); PIFs enhance tissue elongation through auxin signaling to result in a larger leaf area to cool the entire plant [10]. Another downstream acclimatization process involves the expression of heat shock proteins (HSP), reactive oxygen species (ROS) signaling, osmotic regulation, water transport, and cell wall modifications, among others [16].

Thus, transcriptional information suggests that temperature sensing is organ specific, with the autonomy of the root tissue triggering the cell elongation process independently of the upper sections of the plant, while the shoot responds locally and systemically [17]. This implies that the thermal detection mechanism in the roots is different (and not characterized) from PhyB and phototropin signaling because they depend on the variation of light, which is absent in the roots [17].

The above suggests that a lack in the fine thermo-sensing processes could be still unknown in the majority of crops of agricultural importance, such as durum wheat. The present study aims to analyze the physiological and transcriptional response of durum wheat seedlings to conditions of increased temperature (+2 °C). The findings constitute the first insight at the regulation of the mechanism influenced by a temperature increase in wheat, which represents valuable information to improve our understanding of durum wheat adaptation strategies and enhance the development of new heat-tolerant varieties to help farmers in vulnerable regions cope with increasing climate risks.

## 2. Results

The morphometrical assay was carried out to compare the physiological effect of an elevated temperature (+2 °C) vs. the optimal temperature condition for durum wheat production in the Yaqui Valley (28 °C). Thus, a negative significant (Tukey-Kramer test, *p*-value < 0.05) effect on root length (−20%), dry shoot weight (−24.5%), and biovolume index (−28.2%) was observed by elevated temperatures. However, the root:shoot ratio showed a positive significant effect (57.9%) under that treatment (Table 1). On the other hand, no significant differences were observed in the shoot length (−6.7%), and dry root weight (10.8%). However, it was also observed that the dry root weight and root:shoot ratio tended to increase in seedlings under increased temperature (Table 1).

### 2.1. Validation of RNA Extraction from Durum Wheat Seedlings, Quality, and Sequencing

The RNA extraction from whole durum wheat seedlings (root and foliar tissue) had high-quality RNA, 92.73 ± 24.1 ng/µL, for seedlings growing at the optimal temperature (T_optimal), and 109.73 ± 32.4 ng/µL for seedlings under increased temperature (+2 °C) (T_heat). Additionally, all RNA Integrity Number (RIN) values were at 8.0–9.0, which indicates that isolated RNA is suitable for RNA-seq analysis [18].

Six cDNA libraries (two treatments (seedlings growing under optimal vs. increased temperature) x three biological replicates) were sequenced. The high-throughput sequencing yielded 484,576,325 paired-end raw reads, distributed in 222,215,591 and 262,360,734 raw reads for T_optimal and T_heat treatments, respectively. After reads trimming/clipping, 380,019,949 high-quality reads were obtained (78.42% of paired-end raw reads), where 178,519,974 corresponded to T_optimal and 201,499,975 to T_heat (Table S1). Then, 354,652,477 reads (93.32% of total trimmed reads) were mapped to *T. dicoccoides* WEWSeq_v.1.0. genome (166,012,696 and 188,639,781 reads belonged to T_optimal and T_heat treatment, respectively). The uniquely mapped reads were 109,451,851 reads for T_optimal and 130,998,011 reads for T_heat treatments (Table S1).

**Figure 1:**
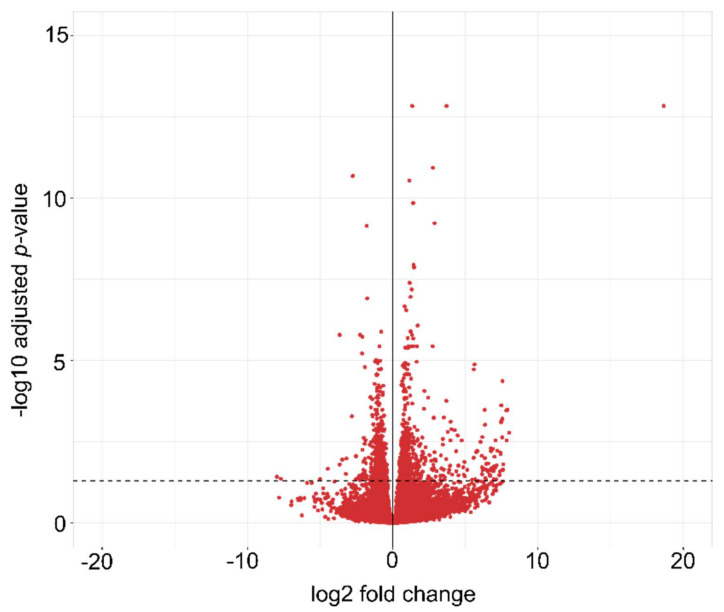
Volcano plot of differentially expressed genes (DEGs) in seedlings (GS21-22) under T_optimal vs. T_heat. Mean log2 fold change is plotted against the −log10 FDR adjusted *p*-values for expressed genes. Genes up of the segmented line indicate differentially expressed genes (DEGs) (FDR ≤ 0.05).

A differential expression analysis between the two studied treatments (T_optimal vs. T_heat) identified 1064 differentially expressed genes (DEGs) (False Discovery Rate (FDR) ≤0.05). After filtering by |log2 fold change| ≥ 1.3, 283 DEGs (78 downregulated and 205 upregulated) were obtained (Figure 1, Table S2). These 283 DEGs were used to perform the enriched gene ontology analysis.

### 2.2. Enriched Gene Ontology Analysis

**Figure 2:**
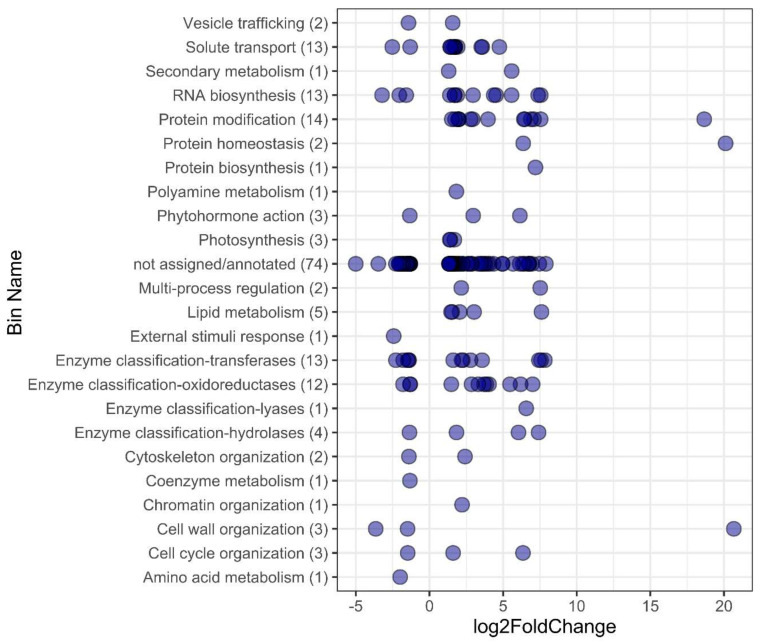
DEGs responsive to an elevated temperature (+2 °C) of durum wheat seedlings, grouped into functional categories according to MapMan4 software and sense of expression (log2FoldChange). Each bubble represents a DEG. In parentheses is the number of DEGs in Bin.

The sense and level of expression into functional categories by Mapman4 [19] showed that those initial 283 DEGs were classified in (i) not assigned/not annotated (106), and (ii) 20 functional categories (including not assigned/annotated) (177). The latter was composed of 47 downregulated genes (DR) and 130 upregulated genes (UR). The functional categories with the highest number of DEGs were Bin 35.1 Not assigned/annotated (74 DEGs (24 DR and 50 UR)); Bin 50 Enzyme classification (30 DEGs (9 DR and 21 UR)); Bin 18 Protein modification (14 UR); Bin 24 Solute transport (13 DEGs (2 DR and 11 UR)); Bin 15 RNA biosynthesis (13 DEGs (3 DR and 10 UR)); and Bin 5 Lipid metabolism (5 UR). Other categories were represented with five DEGs (Bin 1 Photosynthesis (3 UR); Bin 11 Phytohormone action (1 DR and 2UR); and Bin 21 Cell wall organization (2 DR and 1 UR)); and two DEGs (Bin 9 Secondary metabolism (2 UR), Bin 19 Protein homeostasis (2 UR), Bin 20 Cytoskeleton organization (1 DR and 1 UR), and Bin 27 Multi-process regulation (2 UR)) (Figure 2 and Table S3).

**Figure 3:**
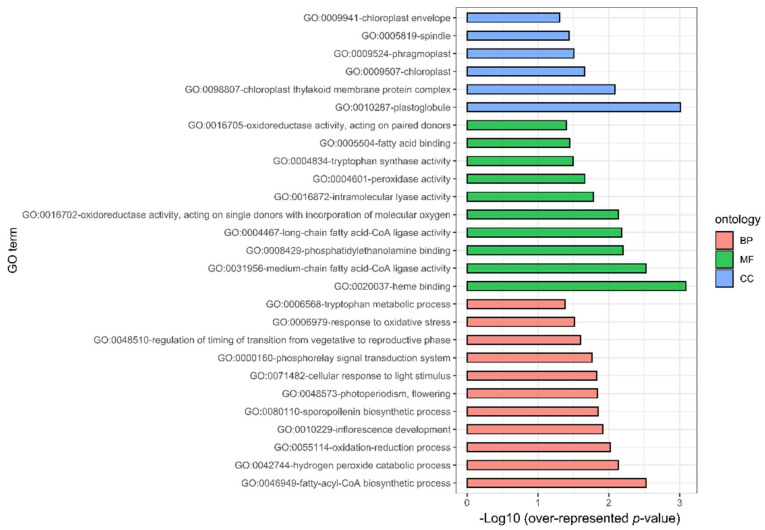
Gene ontology terms overrepresented (FDR < 0.05) in DEGs (FDR ≤ 0.05 and |log2 fold change| ≥ 1.3) of durum wheat seedlings under T_optimal vs. T_heat. Bar color indicates the three categories of GO terms: Biological Processes (BP), Molecular Functions (MF), and Cellular Component (CC).

All obtained significant DEGs (283; FDR ≤ 0.05 and |log2 fold change| ≥ 1.3) were classified according to their relation to biological processes (BP), molecular functions (MF), and cellular component (CC), through a gene ontology (GO) enrichment analysis performed with GOSeq [20]. Thus, 27 GO terms were significantly enriched (overrepresented FDR ≤ 0.05) (i), where (i) eleven belonged to BP, from which the most represented were fatty-acyl-CoA biosynthetic process (GO:0046949, FDR = 2.97 × 10^−3^), hydrogen peroxide catabolic process (GO:0042744, FDR = 7.37 × 10^−3^), oxidation–reduction process (GO:0055114, FDR = 9.52 × 10^−3^), and inflorescence development (GO:0010229, FDR = 0.0122); (ii) six to MF of which the three most represented were the theme-binding terms (GO:0020037, FDR = 8.13 × 10^−4^), medium-chain fatty acid-CoA ligase activity (GO:0031956, FDR = 2.97 × 10^−3^), phosphatidylethanolamine binding (GO:0008429, FDR = 6.30 × 10^−3^), long-chain fatty acid-CoA ligase activity (GO:0004467, FDR = 6.55 × 10^−3^), and oxidoreductase activity, acting on single donors with the incorporation of molecular oxygen (GO:0016702, FDR = 7.29 × 10^−3^); and (iii) ten to CC, where plastoglobule (GO:0010287, FDR = 9.70 × 10^−4^), chloroplast thylakoid membrane protein complex (GO:0098807, FDR = 8.18 × 10^−3^), chloroplast (GO:0009507, FDR = 0.0223), phragmoplast (GO:0009524, FDR = 0.0309), spindle (GO:0005819, FDR = 0.0363), and chloroplast envelope, were the most represented terms (GO:0009941, FDR = 0.0492) (Figure 3 and Table S4).

## 3. Discussion

### 3.1. Morphometrical Influence of An Elevated Temperature

In this study, we observed a significant decrease in several morphometrical traits in plants grown under an increase in temperature, i.e., root length (−20%), dry shoot weight (−24.5%), and biovolume index (−28.2%) (Table 1). This coincides with a significant loss of biomass in maize and broccoli seedlings at a temperature above 30 °C in the vegetative stage, previously reported by Hatfield and Prueger (2015) [21]. Such effects could be attributed to the inhibition of the transport of photoassimilates and nitrogen compounds through plant structures and protein denaturation [10,15].

However, a significant increase of 57.9% in the root:shoot ratio (dry mass) was also observed; this suggests a mobilization and reprogramming of nutrients from the upper tissues to the root system, as an early response of adaptation to the increases in temperature [9,22]. Such behavior was also described by Zhang et al. (2015) [23]. in *A. thaliana* under heat stress of 42 °C to 45 °C, with and without a gradual acclimation process. This gradual increase in temperature maintains or increases the root growth and surviving rate. This strategy has been reported in other *Arabidopsis* accessions under high ambient temperatures, suggesting that root nutrient mobilization is required to move sensitive and active meristematic tissue away from the shallow soil, which absorbs heat and can promote cooling by allowing better access to water [15]. Additionally, adaptation to a high ambient temperature also involves physiological processes, such as photosynthetic acclimation, respiration, carbon balance changes, cell-wall modifications, and reactive oxygen species (ROS) regulation.

### 3.2. Acceleration of Development

The progression of the developmental stage was evidenced by the overrepresentation of ontologies associated with this process, i.e., inflorescence development (GO:0010229 (FDR: 0.0121)), sporopollenin biosynthetic process (GO:0080110 (FDR: 0.0140)), photoperiodism and flowering (GO:0048573 (FDR: 0.0143)), regulation of timing of transition from the vegetative to reproductive phase (GO:0048510 (FDR: 0.0249)), spindle (GO:0005819 (FDR: 0.0363)), and phragmoplast (GO:0009524 (FDR: 0.0309)) (Figure 3). In durum wheat, the stages most sensitive to heat stress are anthesis and the vegetative period; both stages are characterized by being crucial for the acquisition of nutrients (vegetative stage) and the grain filling (anthesis) [11]. Physiologically, the effect of heat stress is presented by a reduced photosynthetic rate, accelerated development, reduced flowering time, and fewer grains per spike [13]. It has been observed that the negative effects on yield may be imperceptible up to an upper limit of 31 °C near the flowering stage. This may depend on the genotype, the availability of water, and the stage of development [14]. This acceleration of plant development has been proposed as an effect of the modification of the circadian clock [24]; some of the signals regulating this mechanism include light and temperature and are associated with the compensation of plant growth in seasonal changes [25]. A study in *A. thaliana* reported that circadian modifications and their physiological effects, such as an accelerated change to the reproductive stage, are more significant in the increase in temperature than in the decrease [26].

### 3.3. Photosynthesis and ATPase Activity

**Figure 4:**
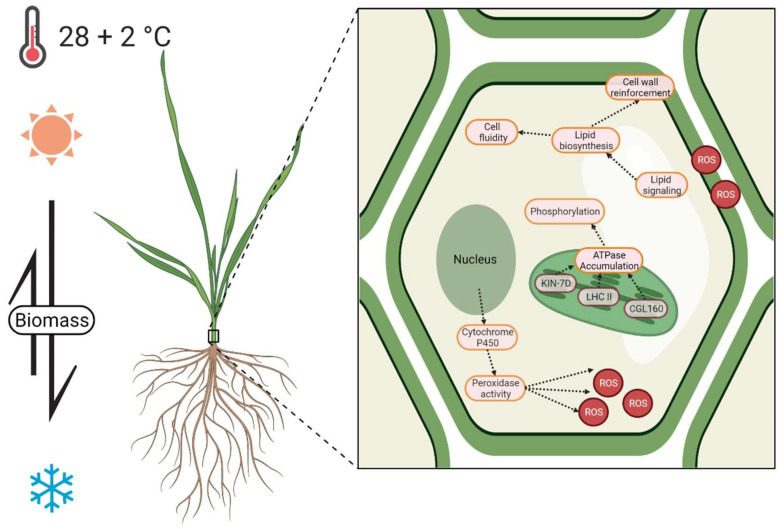
The first insight of the transcriptional changes of durum wheat seedlings under an increase of +2 °C vs. optimal temperature (28 °C), and their possible relationship to morphometric traits. The asymmetric arrow denotes the greater transport of biomass to the roots compared to the shoots as a mechanism of protection and cooling of meristem tissue. The three main molecular acclimatization mechanisms observed are also illustrated: the promotion of photosynthesis through the accumulation of ATPases; lipid biosynthesis to (1) reinforce the cell wall; (2) adjust cell fluidity; and (3) lipid signaling; and peroxidase activity through the expression of cytochrome P450. KIN-7D, Kinesin-like protein; LHC II, light-harvesting complex II; CGL160, conserved only in the green lineage 160; ROS, reactive oxygen species. Created with BioRender.com (accessed on 28 November 2021).

At the transcriptional level, we highlight the DEGs that belong to the ontologies below. The genes in Bin 1 “Photosynthesis” were upregulated (UR), which included the CGL160 factor (TRIDC3BG045570, (FC: 1.689)), the light-harvesting complex LHCb1/2/3 (TRIDC5BG070090 (FC: 1.420)), and Kinesin-like protein KIN-7D (TRIDC7AG021480 (1.375)) (Figure 2 and Figure 4). These genes are related to ATPase activity and have been reported to actively regulate photosynthetic activity under different luminosity conditions [27,28,29,30]. Previous studies in *A. thaliana* have described that the molecular response of the upper sections of plants to thermal increase is highly related to light sensors as such increases usually depend on the luminous intensity [18,24].

On the other hand, photosynthesis activates the phosphorylation and accumulation of ATPase, which have effects on the maintenance of lipid membrane integrity. It has been reported that the overexpression of this process has positive implications for thermal tolerance in *Arabidopsis* [31,32]. The aforementioned is suggested by the overrepresentation of ontologies related to photosynthetic activity and thylakoid membrane to compensate the effect of increases in temperature, such as chloroplast thylakoid membrane protein complex (GO:0098807 (FDR: 0.008)), cellular response to light stimulus (GO:0071482 (FDR: 0.0148)), chloroplast (GO:0009507 (FDR: 0.0219)), chloroplast envelope (GO:0009941 (FDR: 0.0492)), and plastoglobule (GO:0010287 (FC: 0.0009)) (Figure 3). The activation of photosynthesis has been proposed as one of the first responses to thermal sensitivity and carries out the acclimatization process; this activation involves ATPase accumulation, stomatic opening, and light detection (due to the light and heat share sensing mechanism) as strategies to cope with the thermal increase [10]. It is also a requirement to accelerate physiological development to accomplish the biological cycle of plants [33].

### 3.4. Regulation of Lipid Biosynthesis

Another behavior observed in plants at the threshold of thermal stress is the increase in water accumulation through the synthesis of lipids and the reinforcement of the cell wall [34]. High temperatures affect the lipid composition and viscosity of the membrane, the hotter temperature made more fluid cell membranes. This phenomenon is attributed to the activation of the accumulation and remodeling of the lipid composition of the membrane (Figure 4) [35,36].

In this study, this strategy was observed by the regulation of genes in ontologies such as medium-chain fatty acid-CoA ligase activity (GO:0031956 (FDR: 0.0029)), fatty-acyl-CoA biosynthetic process (GO:0046949 (FDR: 0.0029)), and long-chain fatty acid-CoA ligase activity (GO:0004467 (FDR: 0.0065)) (Figure 3). Additionally, the classification by Mapman showed the regulation of Bin 5 Lipid metabolism composed by DEGs 3-ketoacyl-CoA synthase (KCS) (TRIDC5BG049870 (FC: 7.606)), 3-ketoacyl-CoA synthase (KCS) (TRIDC1AG004560 (FC: 3.028)), caleosin (TRIDC2BG056060(FC: 2.049)), isocitrate lyase (TRIDC2BG032830 (FC: 1.539)), stearoyl-ACP desaturase (TRIDC5AG021050 (FC: 1.463)); Bin 35.1 not assigned/annotated, such as fatty-acid-binding protein 1 (TRIDC2BG029870 (FC: 6.126)), UDP-glycosyltransferase 91A1 (TRIDC3BG060650 (FC: 2.259)), GDSL esterase/lipase (TRIDC3AG006590 (FC: 1.513)), GDSL esterase/lipase (TRIDC6BG067850 (FC: 1.332)), and UDP-glucosyltransferase UGT13248 (TRIDC5BG026280 (FC: 3.572)) (Figure 3). These genes have been associated with a common response to oxidative stress due damage to membranes (lipid peroxidation) [37].

### 3.5. ROS Regulation and Detoxification

A well-known effect of abiotic stress in plants, including thermal stress, is the production of ROS, which can eventually oxidize lipids, proteins, and DNA, and hence trigger apoptosis [11,18,38]. To prevent this, plants accumulate antioxidant secondary metabolites such as flavonoids, alkaloids, or terpenoids (isoprenoids), as observed in Bin 9 Secondary metabolism, i.e., aromatic L-amino acid decarboxylase (TRIDC2BG090650 (FC: 5.580)), isopentenyl diphosphate isomerase (TRIDC2BG032150 (FC: 1.305)); Bin 35.1 not assigned/annotated, as phenolic glucoside malonyltransferase 2 (TRIDC4BG008640 (FC: 1.470)); Bin 50 Enzyme classification, as flavanone 3-dioxygenase 2 (TRIDC5AG007500 (FC: 2.854)), phenolic glucoside malonyltransferase 2 (TRIDC4BG008630 (FC: 2.179)), 9-cis-epoxycarotenoid dioxygenase NCED1 (TRIDC6BG052920 (FC: 3.946)), and Geraniol 8-hydroxylase (TRIDC4BG000670 (FC: −1.307)) (Figure 2).

The main strategy that plants use to lessen the effects of oxidative stress is peroxidase activity. This process was highly represented in the ontology enrichment analysis, specifically the hydrogen peroxide catabolic process (GO:0042744 (FDR: 0.0073)), oxidation-reduction process (GO:0055114 (FDR: 0.0095)), peroxidase activity (GO:0004601 (FDR: 0.0217)), response to oxidative stress (GO:0006979 (FDR: 0.0303)), and oxidoreductase activity, acting on paired donors, with the incorporation of or reduction in molecular oxygen (GO:0016705 (FDR: 0.0395)), oxidoreductase activity, acting on single donors with the incorporation of molecular oxygen and incorporation of two atoms of oxygen (GO:0016702 (FDR: 0.0072)) (Figure 3). The DEGs related to ROS regulation and detoxification described by Mapman classification include glutathione S-transferase (TRIDC2BG032750 (FC: 1.513), Bin 18 Protein modification), peroxidase 3 (TRIDC7BG058370 (FC: 1.733), Bin 24 Solute transport), peroxidase 2 (TRIDC6AG056910 (FC: 1.522), Bin 24 Solute transport); Bin 35.1 not assigned/annotated, as peroxidase 70 (TRIDC6BG011930 (FC: 4.093)), peroxidase 2 (TRIDC2BG015880 (FC: 2.039)), and glutathione S-transferase U18 (TRIDC1BG032670 (FC: 1.338)). DEGs were previously reviewed as important elements of ROS production and detoxification process under stress conditions [38]. Furthermore, a group of cytochrome P450 family 294 genes highly expressed during chronic thermal increase was observed, including TRIDC6BG046780 ((FC: 4.521) Bin 15 RNA biosynthesis), TRIDC6BG026600 ((FC: 6.773) Bin 35.1 not assigned/annotated), TRIDC2AG019170 ((FC: 6.187) Bin 50 Enzyme classification), TRIDC2BG010230 ((FC: 5.467) Bin 50 Enzyme classification), and TRIDC2AG008470 ((FC: 3.316) Bin 50 Enzyme classification). These genes exhibit peroxidase activity under abiotic stress, such as drought, heat, oxygen and cold [9,37,39] (Figure 3 and Figure 4).

Additionally, the influence of post-transcriptional regulation through non-coding RNAs (miRNA, siRNA, lncRNA) is well known. It has been reported that the target genes of such RNAs are generally those with ontologies related to cell wall architecture, ROS regulation, and the development of the next phase, among others [40,41]. Similarly, epigenetic mechanisms have been found to be involved in the response to thermal stress, such as DNA methylation, histone modifications, and chromatin remodeling [42]. For the above, the struggles to fully decipher the response to thermal stress will encompass various levels of molecular organization.

## 4. Materials and Methods

### 4.1. Plant Material and Growth Conditions

Seeds of durum wheat (*Triticum turgidum* subsp. *durum*) cv. CIRNO C2008 were selected due to their wide use in the Yaqui Valley, Cd. Obregon, Sonora, México [43]. Wheat seeds were surface sterilized according to Robles Montoya et al. (2020) [44] using 0.1% sodium hypochlorite (30 s), washed twice with sterilized distilled water (30 s), and incubated for five days at room temperature (25 °C) until germination. Subsequently, the seedlings were placed in sterilized and hermetic containers (washed with ethanol at 70% *v/v* and treatment with ultraviolet light for 1.5 h) with 130 g of an autoclaved substrate (30 min, 121 °C, 15 lbs in three 24 h intervals) (soil (40%): perlite (60%)) [45]. The soil used in this work was previously characterized according to Verhulst et al. (2009) [46] and Valenzuela-Aragon et al. (2019) [47]; such soil showed a high clay content (50 ± 10%), low organic matter content (0.79 ± 0.05%), and an electrical conductivity (CE) value of 1.8 ± 0.1 dS m^−1^, and a slightly alkaline pH value (7.74 ± 0.05). Additionally, this soil showed a saturation percentage of 48 ± 1.73%, CEC of 38.7 ± 3.0 meq/100 g, and a medium-high content of macro-elements (nitrogen (32 ± 8.21 ppm), phosphorus (10.4 ± 0.89 ppm), calcium (5746.6 ± 473.9 ppm), magnesium (523.3 ± 71.2 ppm), potassium (613.3 ± 30.5 ppm), sodium (920 ± 20 ppm)) and microelements (Fe (5.16 ± 0.33 ppm), Mn (10.03 ± 1.41 ppm), Zn (0.4 ± 0.06 ppm), and Cu (1.0 ± 0.03 ppm)). For irrigation purposes, to be more similar to field conditions, 70 mL of distilled and sterilized water was added once according to the soil moisture recommendations for the used durum wheat variety [43].

Two treatments were evaluated (*n* = 66 seedlings per treatment (21 for morphometric assay and 15 to RNASeq (in triplicate)): (i) seedlings grown at optimal temperature conditions (28 °C days and 15 °C night) for durum wheat production in the Yaqui Valley, called “T_optimal” and (ii) seedlings grown under conditions of an elevated temperature (+2 °C) compared to the optimal conditions (30 °C days and 17 °C night), called “T_heat”. These seedlings growing in sterilized hermetic containers were placed in a growth chamber (Biobase #BJPX-A450, Shandong, China) with the following parameters according to durum wheat requirements: photoperiod day/night 14:10 for 45 days (development stage GS21-22) [48,49].

### 4.2. RNA Extraction and RNA-Seq Analysis

Fifteen seedlings were harvested per treatment (in triplicate) and frozen using liquid nitrogen. The samples were then ground in a DEPC-treated mortar and stored at −80 °C until processing [50]. Total RNA was isolated using a modified TRIzol^®^ (Life Technologies; ThermoFisher, Waltham, MA, USA) method described by Chaparro-Encinas et al. 2020 [51]. The extracted total RNA was treated with DNase I (Ambion™) to remove genomic DNA according to the manufacturer’s instructions, and subsequently, RNA Integrity Number (RIN) was obtained using a BioAnalyzer Agilent 2100 (Agilent Technologies, Santa Clara, CA, USA) to evaluate their suitability for downstream applications. The total RNA samples with RIN >8 were considered suitable for library preparation [18], with the TruSeq Stranded Total RNA Library Kit (Illumina, San Diego, CA, USA), and sequenced by Illumina^®^ 2 × 150 en el NextSeq 500 platform (2 × 150 bp). A yield of approximately 85 million paired-end reads per library was obtained.

The quality of raw reads was examined using the *FastQC* (http://www.bioinformatics.babraham.ac.uk/projects/fastqc (accessed on 1 August 2021)) tool before and after the trimming process. Raw reads were filtered with *Trimmomatic* 0.36 [52] by using the parameters SLIDINGWINDOW:4:30 and MINLEN:50 to deplete TruSeq adapters, low-quality reads, and short sequences (<50 bp). Trimmed reads were mapped to the reference genome of *Triticum dicoccoides* “Zavitan” WEWSeq_v.1.0 [53], which is the closest ancestor of durum wheat by using default parameters in HISAT_HEAT [54]. Then, transcript abundances were counted with HTSeq-count [55] using the annotation file (Triticum_dicoccoides.WEWSeq_v.1.0.43). Thus, only unambiguously mapped reads were selected for further analysis.

Transcript abundance counts were used for gene-level differential expression analysis using the DESeq2 package [56]. A principal component analysis (PCA) was performed with DESeq2 function to reproducibility and biological variations among samples purposes. Differentially expressed genes (DEGs) were defined as those with adjusted *p*-value (False Discovery Rate, FDR) <0.05 and a fold change of at ± 1.3. Subsequently, DEGs were annotated with Mercator4 and visualized with MapMan4 [19]. Gene identifiers and Log2 fold change values of DEGs were imported into the MapMan4 framework, and transcripts were assigned into Bins.

To enriched-gene ontology analysis, all gene ontology (GO) terms associated with DEGs were extracted from the BioMart database [57]. Then, the R package GOseq [20] was used to perform a gene length bias correction with Wallenius non-central hypergeometric distribution. Only those GO terms with overrepresented FDR <0.05 were considered as significantly enriched.

### 4.3. Statistical Analysis

For durum wheat morphometric data (shoot and root length, dry weight of shoots and roots, and biovolume index (stem circumference x shoot length) the statistical analysis carried out was ANOVA and Tukey–Kramer range test (*p* < 0.05) [50]. This consisted of two treatments (*n* = 21 seedlings per treatment): (i) seedlings grown at optimal temperature conditions (28 °C days and 15 °C night) for durum wheat production in the Yaqui Valley and (ii) seedlings grown under conditions of an elevated temperature (+2 °C) compared to the optimal conditions (30 °C days and 17 °C night).

On the other hand, all statistical analyses for RNA-Seq data were carried out according to the software and parameters previously mentioned in Section 4.2. RNA extraction and RNA-Seq analysis.

## 5. Conclusions

The main observed response of durum wheat seedlings to the increase in temperature (30 °C vs. 28 °C) was the transcriptional regulation of photosynthesis and ATPase activity; the biosynthesis and remodeling of lipid composition to reinforce the cell wall, water content, and modulate membrane fluidity; and ROS regulation as a signaling and detoxification process induced by thermal stress. These transcription patterns showed physiological signs in the acceleration of phenological progression to the reproductive stage and reprogramming of nutrient mobilization for root development, such patterns suggest a growth-to-escape strategy. These findings complement the state of the art on molecular mechanisms in plants to reduce or even tolerate the impact of climate change.

The arguments presented in this study are based on the observed transcriptomic patterns and constitute the first insights into the mechanisms regulated by the increase in temperature. Therefore, it is necessary to deepen the mechanisms proposed through multi-disciplinary approaches, including qPCR, proteomics, post-transcriptional studies (non-coding RNAs), phenomics, and epigenomics.

## Figures and Tables

**Table 1:** Morphometrical effect of durum wheat seedlings under increased temperature conditions (+2 °C) in a growth chamber.

Biometrical Traits	Optimal Temperature (28 °C)	Increased Temperature (30 °C)	Difference (%)
Shoot length (cm)	22.6 ± 2.9	21.1 ± 4.9	−6.73%
Root length (cm)	16.6 ± 3.6 *	13.2 ± 5.0	−20.00%
Dry shoot weight (g)	38.0 ± 7.1 *	28.7 ± 10.3	−24.50%
Dry root weight (g)	52.9 ± 12.3	58.6 ± 21.6	10.80%
Root:shoot ratio (dry mass, g)	1.5 ± 0.5	2.4 ± 1.4 *	57.92%
Biovolume index	130.8 ± 9.2 *	93.9 ± 29.2	−28.20%

Means (*n* = 21) ± standard deviation with an asterisk (*) are significantly different (increased vs. optimal temperature treatments), according to the Tukey-Kramer test (*p* < 0.05).

## Data Availability

The datasets generated during and/or analyzed during the current study are available in the SRA from the NCBI repository under accession number PRJNA780180 (https://www.ncbi.nlm.nih.gov/bioproject/PRJNA780180) (Accessed on 9 December 2021).

## Supplementary Materials

The following are available online at https://www.mdpi.com/article/10.3390/plants10122792/s1, Table S1. Distribution of paired-end reads after trimming and mapping process for T_optimal and T_heat seedlings, Table S2. List of DEGs filtered by adjusted FDR ≤ 0.05, and |log2 fold change| ≥ 1.3, Table S3 Functional classification of DEGs [FDR ≤ 0.05 and |log2 fold change| ≥ 1.3] by Mercator/Mapman4, Table S4. Gene ontology enrichment analysis by R package GOSeq, significantly enriched (FDR ≤ 0.05).
