# Supplementary material for: Transcriptional Regulation of Metabolic and Cellular Processes in Durum Wheat (Triticum turgidum subsp. durum) in the Face of Temperature Increasing
Source: Plants (Basel). 2021 Dec 16;10(12):2792. doi: 10.3390/plants10122792 (PMC8703274; doi:10.3390/plants10122792)
Supplement: Supplementary file 1 [file plants-10-02792-s001.zip › plants-1486717-supplementary.pdf]

Supplementary material

Transcriptional Regulation of Metabolic and Cellular Processes in Durum Wheat (*Triticum turgidum* subsp. *durum*) in the Face of Temperature Increasing

Luis Abraham Chaparro-Encinas, Gustavo Santoyo, Juan José Peña-Cabriales, Luciano Castro-Espinoza, Fannie Isela Parra-Cota, and Sergio de los Santos-Villalobos

Table S1. Distribution of paired-end reads after trimming and mapping process for T_optimal and T_heat seedlings

Samples	Paired-ends raw reads	Trimmed Reads (*)	Overall mapped reads (**)	Multimapped reads (**)	Uniquely mapped reads (**)
T_optimal (1)	94,598,223	78,015,984 (82.47%)	71,704,491 (91.91%)	9,581,229 (12.28%)	50,520,079 (64.76%)
T_optimal (2)	52,091,756	41,741,296 (80.13%)	38,965,500 (93.35%)	6,915,879 (16.57%)	23,683,689 (56.74%)
T_optimal (3)	75,525,612	58,762,694 (77.80%)	55,342,705 (94.18%)	8,123,507 (13.82%)	35,248,083 (59.98%)
T_heat (1)	85,038,673	63,715,241 (74.93%)	58,802,796 (92.29%)	2,757,984 (4.33%)	51,781,928 (81.27%)
T_heat (2)	93,930,810	74,102,477 (78.89%)	70,115,764 (94.62%)	11,254,431 (15.19%)	44,236,107 (59.70%)
T_heat (3)	83,391,251	63,682,257 (76.37%)	59,721,221 (93.78%)	13,345,674 (20.96%)	34,979,976 (54.93%)

* The percentage value was calculated for paired-end raw reads. ** The percentage value was calculated

Table S2. List of DEGs filtered by adjusted FDR ≤ 0.05, and |log2 fold change| ≥ 1.3

Gene ID	log2 fold change	FDR
TRIDC5AG047410	-4.352	9.23E-29
TRIDC4AG063850	3.597	7.10E-19
TRIDC6AG005710	-18.658	1.46E-13
TRIDC2AG065830	-3.701	1.46E-13
TRIDC3AG019850	-1.341	1.46E-13
TRIDC4BG032750	-2.765	1.17E-11
TRIDC4AG063860	2.751	2.08E-11
TRIDC6AG058030	-1.407	1.42E-10
TRIDC5BG009760	-2.882	5.93E-10
TRIDC6BG054630	1.789	7.12E-10
TRIDC5AG046840	-1.436	1.14E-08
TRIDC2AG043690	-1.477	1.36E-08
TRIDC2BG032150	-1.305	6.49E-08
TRIDC6BG017260	1.757	1.22E-07
TRIDC6BG043940	-1.715	8.36E-07
TRIDC6BG067850	-1.332	1.62E-06
TRIDC5BG018270	2.243	1.62E-06
TRIDC4AG063880	3.652	1.62E-06
TRIDC7AG075020	2.081	1.86E-06
TRIDC5BG070090	-1.420	2.11E-06
TRIDC7AG022250	-2.746	3.63E-06
TRIDC2BG046370	-1.414	3.63E-06
TRIDC6AG041660	-1.679	3.69E-06
TRIDC6AG011890	2.103	6.03E-06
TRIDC6AG010840	-1.644	1.09E-05
TRIDC3BG052560	-5.628	1.32E-05
TRIDC7AG033730	1.917	1.59E-05
TRIDC2BG090650	-5.580	1.87E-05
TRIDC4BG042950	-7.556	4.29E-05
TRIDC4BG008630	-2.179	8.64E-05
TRIDC7AG006240	1.541	1.33E-04
TRIDC7BG061660	-2.432	1.38E-04
TRIDC2AG001630	1.397	1.55E-04
TRIDC5AG020540	-3.690	1.74E-04
TRIDC3BG025030	-1.346	2.10E-04
TRIDC3BG043350	-7.475	2.39E-04
TRIDC7BG056330	1.507	2.77E-04
TRIDC5BG051120	-2.157	3.06E-04

TRIDC6BG072880	-7.903	3.23E-04
TRIDC7AG021890	-6.328	3.29E-04
TRIDC7BG074740	-7.826	3.45E-04
TRIDC2BG042360	-20.670	4.24E-04
TRIDC2BG006380	1.399	4.76E-04
TRIDC2BG038880	2.809	5.18E-04
TRIDC5BG053460	1.376	5.23E-04
TRIDC7BG045270	-3.522	5.66E-04
TRIDC2AG077130	-2.826	5.77E-04
TRIDC5BG010470	-7.548	6.11E-04
TRIDC7AG013410	-7.519	6.72E-04
TRIDC2BG059360	-20.112	7.17E-04
TRIDC3AG017450	-3.988	7.65E-04
TRIDC2BG080020	-7.458	7.91E-04
TRIDC7BG021730	1.312	9.17E-04
TRIDC4AG012300	1.339	9.24E-04
TRIDC5AG043950	-6.355	9.47E-04
TRIDC2AG041570	-1.491	1.10E-03
TRIDC6BG025800	-4.045	1.30E-03
TRIDC6AG021140	-4.271	1.39E-03
TRIDC3BG045570	-1.689	1.39E-03
TRIDC7AG062880	-3.786	1.58E-03
TRIDC2BG068510	-8.010	1.67E-03
TRIDC1BG040170	-1.321	1.81E-03
TRIDC2BG051270	-4.468	2.02E-03
TRIDC6BG007940	-6.172	2.26E-03
TRIDC3AG012180	-7.534	2.32E-03
TRIDC2BG015880	-2.039	2.34E-03
TRIDC5AG029700	-1.410	2.35E-03
TRIDC4BG037100	1.992	2.38E-03
TRIDC7AG031090	-3.451	2.61E-03
TRIDC4AG023830	-1.727	2.61E-03
TRIDC1BG004790	-1.720	2.72E-03
TRIDC2AG066940	1.322	2.72E-03
TRIDC2BG025970	-7.080	2.85E-03
TRIDC2AG023840	1.870	2.85E-03
TRIDC3AG040710	-3.977	2.87E-03
TRIDC2BG034440	-7.837	2.88E-03
TRIDC2AG055510	-4.746	2.88E-03
TRIDC1BG031820	-1.671	2.97E-03
TRIDC5AG021050	-1.463	2.98E-03

TRIDC1AG037370	-1.443	3.06E-03
TRIDC5BG011000	-6.045	3.17E-03
TRIDC6AG011900	1.475	3.20E-03
TRIDC3AG055380	-1.477	3.66E-03
TRIDC2AG033960	1.927	3.66E-03
TRIDC5BG036930	-1.918	3.68E-03
TRIDC3AG009740	-3.452	3.86E-03
TRIDC2BG082330	-1.877	3.86E-03
TRIDC3BG039950	1.562	3.91E-03
TRIDC5BG058740	1.580	3.96E-03
TRIDC7AG052900	-1.825	4.22E-03
TRIDC2BG061970	1.392	4.44E-03
TRIDC6BG048590	-2.270	4.63E-03
TRIDC6AG021100	-6.762	4.99E-03
TRIDC5BG000800	-6.904	5.23E-03
TRIDC6AG039210	-1.399	5.45E-03
TRIDC7AG021480	-1.375	5.61E-03
TRIDC1BG040780	2.076	5.61E-03
TRIDC3AG046290	-7.197	5.92E-03
TRIDC4BG052890	-5.821	6.03E-03
TRIDC2BG082650	-6.762	6.09E-03
TRIDC5AG035000	-4.364	6.19E-03
TRIDC2BG056060	-2.049	6.30E-03
TRIDC4BG055730	-2.877	6.60E-03
TRIDC1BG051340	-5.755	6.60E-03
TRIDC1AG061460	-7.408	6.70E-03
TRIDC5BG009120	-2.097	7.33E-03
TRIDC4AG016130	-1.422	7.33E-03
TRIDC2BG039440	-1.418	7.33E-03
TRIDC5BG013060	1.397	7.46E-03
TRIDC2AG080440	-7.432	7.56E-03
TRIDC3AG006590	-1.513	7.57E-03
TRIDC7AG025280	-2.536	8.17E-03
TRIDC2AG008470	-3.316	8.33E-03
TRIDC4AG038540	-2.006	8.33E-03
TRIDC2BG029870	-6.126	8.56E-03
TRIDC5AG068850	2.528	8.77E-03
TRIDC5AG005640	-1.618	8.91E-03
TRIDC5BG026880	-7.003	9.10E-03
TRIDC2AG013320	-1.350	9.61E-03
TRIDC1AG061690	1.652	9.61E-03

TRIDC5BG037960	-5.573	9.84E-03
TRIDC3BG060650	-2.259	1.00E-02
TRIDC7BG053890	-2.212	1.00E-02
TRIDC5AG037990	-6.349	1.01E-02
TRIDC4AG019030	-2.001	1.03E-02
TRIDC7BG019460	3.188	1.03E-02
TRIDC3BG024680	1.481	1.05E-02
TRIDC7AG020140	1.908	1.06E-02
TRIDC3AG040110	-3.682	1.08E-02
TRIDC3BG013490	-2.935	1.10E-02
TRIDC7BG046050	-1.599	1.10E-02
TRIDC4AG008190	-2.884	1.12E-02
TRIDC7BG010170	3.475	1.12E-02
TRIDC5BG006190	1.397	1.21E-02
TRIDC7AG037590	-2.658	1.21E-02
TRIDC6BG021850	-2.265	1.21E-02
TRIDC4BG056890	-1.754	1.21E-02
TRIDC1BG049170	-1.870	1.22E-02
TRIDC6BG071510	-2.968	1.24E-02
TRIDC7AG072970	2.033	1.29E-02
TRIDC6BG073190	-4.942	1.33E-02
TRIDC3BG006400	1.843	1.44E-02
TRIDC7AG005860	-1.729	1.45E-02
TRIDC6BG012300	-6.570	1.47E-02
TRIDC1BG025800	-1.522	1.51E-02
TRIDC6AG042450	-6.454	1.51E-02
TRIDC5BG010520	-2.159	1.53E-02
TRIDC6AG000240	-1.610	1.53E-02
TRIDC5AG014710	-1.541	1.53E-02
TRIDC2BG006310	1.495	1.53E-02
TRIDC2BG087400	-1.575	1.54E-02
TRIDC1AG040150	-1.668	1.54E-02
TRIDC5BG049870	-7.606	1.56E-02
TRIDC2BG010910	3.653	1.59E-02
TRIDC2BG032830	-1.539	1.59E-02
TRIDC3AG022790	-1.785	1.59E-02
TRIDC6AG034550	-2.796	1.62E-02
TRIDC4BG000550	-1.480	1.67E-02
TRIDC5BG078550	-7.149	1.69E-02
TRIDC6BG046780	-4.521	1.72E-02
TRIDC6BG052920	-3.946	1.72E-02

TRIDC7AG019910	-1.833	1.80E-02
TRIDC1BG007600	-1.529	1.81E-02
TRIDC3AG017410	-1.384	1.81E-02
TRIDC6AG014100	-6.982	1.81E-02
TRIDC4BG010840	-1.827	1.84E-02
TRIDC3AG050160	-6.142	1.92E-02
TRIDC7AG045430	-4.859	1.92E-02
TRIDC7AG075040	1.482	1.94E-02
TRIDC7BG032770	-1.738	1.95E-02
TRIDC3BG001230	-7.048	2.02E-02
TRIDC5AG072330	-1.556	2.05E-02
TRIDC4BG000670	1.307	2.06E-02
TRIDC1BG034560	-6.794	2.08E-02
TRIDC5AG065090	-3.356	2.08E-02
TRIDC5AG007500	-2.854	2.08E-02
TRIDC6BG026600	-6.773	2.10E-02
TRIDC3BG037750	1.479	2.12E-02
TRIDC2AG066680	4.473	2.14E-02
TRIDC3AG072040	-1.664	2.14E-02
TRIDC1BG032670	-1.338	2.14E-02
TRIDC2AG077740	-6.280	2.15E-02
TRIDC2BG085700	-2.704	2.19E-02
TRIDC7AG060170	-7.578	2.23E-02
TRIDC1AG043930	-3.888	2.29E-02
TRIDC2AG080650	-3.516	2.29E-02
TRIDC4AG016330	-5.127	2.32E-02
TRIDC5AG007720	-1.704	2.32E-02
TRIDC6AG054990	2.009	2.33E-02
TRIDC2BG048610	-7.604	2.33E-02
TRIDC5BG001040	-6.467	2.34E-02
TRIDC2BG089510	-2.784	2.35E-02
TRIDC2AG019170	-6.187	2.36E-02
TRIDC7AG016880	-6.708	2.37E-02
TRIDC7BG060470	1.322	2.46E-02
TRIDC3BG039900	-6.957	2.50E-02
TRIDC1AG004560	-3.028	2.51E-02
TRIDC5AG037820	-6.885	2.52E-02
TRIDC1BG066730	1.339	2.53E-02
TRIDC7AG020710	1.357	2.53E-02
TRIDC1BG056420	1.908	2.53E-02
TRIDC2BG011890	-4.384	2.57E-02

TRIDC5BG072370	-4.375	2.57E-02
TRIDC7BG003240	-7.382	2.61E-02
TRIDC3AG037860	-6.566	2.62E-02
TRIDC2AG049210	-1.603	2.63E-02
TRIDC7AG075460	1.511	2.63E-02
TRIDC4AG030140	-2.055	2.64E-02
TRIDC1AG041860	-1.316	2.73E-02
TRIDC5BG008940	-3.858	2.75E-02
TRIDC7BG058370	-1.733	2.94E-02
TRIDC6AG056910	-1.522	3.00E-02
TRIDC7BG036190	2.050	3.00E-02
TRIDC2AG023120	3.222	3.08E-02
TRIDC7AG012250	-1.936	3.13E-02
TRIDC1BG016990	-6.152	3.15E-02
TRIDC6BG011930	-4.093	3.25E-02
TRIDC2AG078730	1.360	3.25E-02
TRIDC4BG005240	1.921	3.25E-02
TRIDC5BG082230	-1.705	3.30E-02
TRIDC4AG063010	-4.897	3.30E-02
TRIDC2AG078690	1.862	3.30E-02
TRIDC3AG030320	-6.673	3.32E-02
TRIDC5AG001540	1.328	3.35E-02
TRIDC5AG030910	-7.344	3.36E-02
TRIDC5BG007180	-4.532	3.41E-02
TRIDC4BG008640	-1.470	3.41E-02
TRIDC4AG071950	-6.659	3.41E-02
TRIDC6BG023580	-1.977	3.43E-02
TRIDC2BG051790	-3.467	3.43E-02
TRIDC5BG031060	-1.886	3.43E-02
TRIDC7AG059380	2.280	3.44E-02
TRIDC1AG023310	-6.723	3.50E-02
TRIDC2BG014330	-6.725	3.51E-02
TRIDC2BG032750	-1.513	3.53E-02
TRIDC2BG017450	-3.289	3.54E-02
TRIDC6BG012240	2.280	3.54E-02
TRIDC5AG043470	-3.267	3.57E-02
TRIDC1AG039360	1.776	3.58E-02
TRIDC2AG080710	-2.463	3.61E-02
TRIDC7BG068600	1.402	3.64E-02
TRIDC7BG002180	-4.966	3.67E-02
TRIDC7BG026890	1.503	3.67E-02

TRIDC1BG021020	1.351	3.68E-02
TRIDC7BG036280	-3.536	3.71E-02
TRIDC4AG011220	-2.954	3.71E-02
TRIDC5AG040580	7.976	3.78E-02
TRIDC2BG000740	1.798	3.82E-02
TRIDC6AG003780	-3.582	3.88E-02
TRIDC5AG003240	2.015	3.88E-02
TRIDC6AG000750	1.329	4.09E-02
TRIDC7BG032720	1.347	4.10E-02
TRIDC5BG079600	-6.364	4.12E-02
TRIDC1BG002730	-1.921	4.20E-02
TRIDC6BG021650	1.426	4.22E-02
TRIDC4BG000470	1.304	4.24E-02
TRIDC1BG044360	2.432	4.24E-02
TRIDC2BG004420	2.060	4.27E-02
TRIDC2BG031640	1.489	4.34E-02
TRIDC3AG004470	-1.343	4.34E-02
TRIDC3BG004760	7.708	4.35E-02
TRIDC2AG057350	-2.410	4.35E-02
TRIDC5AG054280	-2.661	4.44E-02
TRIDC1AG037530	1.782	4.45E-02
TRIDC1BG043540	-1.722	4.48E-02
TRIDC4AG065770	5.008	4.48E-02
TRIDC5AG006390	-6.953	4.48E-02
TRIDC1BG042910	-5.678	4.51E-02
TRIDC5BG010450	-6.407	4.55E-02
TRIDC2BG043990	-7.127	4.58E-02
TRIDC7BG045810	2.565	4.59E-02
TRIDC4BG040200	1.300	4.61E-02
TRIDC7BG064160	1.581	4.61E-02
TRIDC2BG010230	-5.467	4.61E-02
TRIDC4BG060190	-2.584	4.61E-02
TRIDC5BG026280	-3.572	4.61E-02
TRIDC1AG047410	-7.092	4.68E-02
TRIDC7BG019110	-3.286	4.77E-02
TRIDC6BG038180	-1.652	4.78E-02
TRIDC4BG060480	2.281	4.78E-02
TRIDC2AG036110	-1.406	4.78E-02
TRIDC2BG014720	-1.480	4.81E-02
TRIDC1AG026860	1.313	4.88E-02

Table S3 Functional classification of DEGs [FDR ≤ 0.05 and |log2 fold change| ≥ 1.3] by Mercator/Mapman4

Gene ID	BIN	Name	Description	log2 Fold change
TRIDC5BG070090	1.1.1.1.1	Photosynthesis/photophosphorylation/photosystem II/LHC-II complex/component LHCb1/2/3	component LHCb1/2/3 of LHC-II complex (original description: none)	1.420
TRIDC3BG045570	1.1.9.3.1	Photosynthesis/photophosphorylation/ATP synthase complex/assembly/assembly factor (CGL160)	CGL160 factor involved in ATP synthase complex assembly (original description: none)	1.689
TRIDC7AG021480	1.2.1.2.4	Photosynthesis/calvin cycle/ribulose-1,5-bisphosphat carboxylase/oxygenase (RuBisCo) activity/RuBisCo assembly/assembly factor (RbcX)	Kinesin-like protein KIN-7D, chloroplastic OS=Oryza sativa subsp. japonica (sp|q6yz52|kn7d_orysj : 268.0)	1.375
TRIDC4BG037100	4.1.5.3.5.1	Amino acid metabolism/biosynthesis/shikimate family/tryptophan/tryptophan synthase heterodimer/subunit alpha	subunit alpha of tryptophan synthase complex (original description: none)	-1.992
TRIDC1AG004560	5.1.6.1.1	Lipid metabolism/fatty acid biosynthesis/endoplasmic reticulum-localized fatty acid elongase (FAE) system/fatty acid elongation complex/3-ketoacyl-CoA synthase (KCS)	3-ketoacyl-CoA synthase (KCS) (original description: none)	3.028
TRIDC5BG049870	5.1.6.1.1	Lipid metabolism/fatty acid biosynthesis/endoplasmic reticulum-localized fatty acid elongase (FAE) system/fatty acid elongation complex/3-ketoacyl-CoA synthase (KCS)	3-ketoacyl-CoA synthase (KCS) (original description: none)	7.606

TRIDC5AG021050	5.1.7.1	Lipid metabolism/fatty acid biosynthesis/fatty acid desaturation/stearoyl-ACP desaturase	stearoyl-ACP desaturase (original description: none)	1.463
TRIDC2BG032830	5.7.3.6.3	Lipid metabolism/lipid degradation/fatty acid degradation/glyoxylate cycle/isocitrate lyase	isocitrate lyase (original description: none)	1.539
TRIDC2BG056060	5.9.3	Lipid metabolism/lipid bodies-associated activities/caleosin lipid body surface protein (CLO/PXG)	caleosin (original description: none)	2.049
TRIDC6AG000750	7.12.6.7.6	Coenzyme metabolism/tetrapyrrol biosynthesis/chlorophyll metabolism/chlorophyll breakdown/red chlorophyll catabolite reductase (RCCR)	red chlorophyll catabolite reductase (RCCR) (original description: none)	-1.329
TRIDC7AG052900	8.5.2.1	Polyamine metabolism/polyamine degradation/FAD-dependent polyamine oxidase activities/cytosolic polyamine oxidase (PAO1)	cytosolic polyamine oxidase (PAO1)	1.825
TRIDC2BG032150	9.1.1.7	Secondary metabolism/terpenoids/mevalonate pathway/isopentenyl diphosphate isomerase	isopentenyl diphosphate isomerase (original description: none)	1.305
TRIDC2BG090650	9.3.1.1	Secondary metabolism/alkaloids/indole alkaloid biosynthesis/aromatic L-amino acid decarboxylase	aromatic L-amino acid decarboxylase (original description: none)	5.580
TRIDC3AG050160	11.1.2.1.1.1	Phytohormone action/abscisic acid/perception and signalling/PYR/PYL-receptor/cytoplasm-localized receptor complex/receptor component PYL/RCAR	receptor component PYL/RCAR of cytoplasm-localized abscisic acid receptor complex (original description: none)	6.142

TRIDC1BG066730	11.10.1.4.1	Phytohormone action/signalling peptides/NCRP (non-cysteine-rich-peptide) category/CIF-peptide activity/CIF-precursor polypeptide	CIF precursor polypeptide (original description: none)	-1.339
TRIDC6BG071510	11.4.2.5	Phytohormone action/cytokinin/perception and signal transduction/A-type ARR response negative regulator	A-type ARR response negative regulator	2.968
TRIDC7BG053890	12.1.2	Chromatin organization/histones/histone (H2A)	Glucan endo-1,3-beta-glucosidase 13 OS=Arabidopsis thaliana (sp|q9fju9|e1313_arath : 298.0) & Enzyme classification.EC_3 hydrolases.EC_3.2 glycosylase(50.3.2 : 296.7) (original description: none)	2.212
TRIDC5AG037990	13.1.1.3.1	Cell cycle organization/cell cycle control/cyclin-dependent regulation/cyclin-dependent protein kinase inhibitor activities/inhibitor (KRP/ICK)	KRP/ICK-type cyclin-dependent kinase inhibitor (original description: none)	6.349
TRIDC3BG037750	13.3.3.2.1	Cell cycle organization/mitosis and meiosis/chromosome segregation/kinetochore proteins/kinetochore assembly protein (CENP-C)	kinetochore assembly protein (CENP-C) (original description: none)	-1.479
TRIDC2AG049210	13.3.6.5.1.7	Cell cycle organization/mitosis and meiosis/meiotic recombination/meiotic crossover/class I interference-sensitive crossover pathway/pachytene checkpoint protein (PCH2/CRC1)	meiotic crossover pachytene checkpoint protein (PCH2/CRC1) (original description: none)	1.603

TRIDC5BG058740	15.3.3.1.6	RNA biosynthesis/RNA polymerase II-dependent transcription/MEDIATOR transcription co-activator complex/head module/component MED19	component MED19 of head module of MEDIATOR transcription co-activator complex (original description: none)	-1.580
TRIDC3AG017410	15.5.1.1	RNA biosynthesis/transcriptional regulation/C2C2 transcription factor superfamily/transcription factor (BBX-DBB)	transcription factor (BBX-DBB) (original description: none)	1.384
TRIDC7BG036190	15.5.14	RNA biosynthesis/transcriptional regulation/transcription factor (MADS/AGL)	protein kinase (RIO) (original description: none)	-2.050
TRIDC6BG046780	15.5.14	RNA biosynthesis/transcriptional regulation/transcription factor (MADS/AGL)	Cytochrome b5 isoform E OS=Arabidopsis thaliana (sp|q42342|cyb5e_arath : 88.6)	4.521
TRIDC7BG003240	15.5.14	RNA biosynthesis/transcriptional regulation/transcription factor (MADS/AGL)	ABC transporter C family member 10 OS=Arabidopsis thaliana (sp|q9lys2|ab10c_arath : 195.0)	7.382
TRIDC4BG042950	15.5.14	RNA biosynthesis/transcriptional regulation/transcription factor (MADS/AGL)	transcription factor (MADS/AGL) (original description: none)	7.556
TRIDC2AG023120	15.5.15	RNA biosynthesis/transcriptional regulation/transcription factor (C2H2-ZF)	C2H2 zinc finger transcription factor (original description: none)	-3.222
TRIDC5AG035000	15.5.15	RNA biosynthesis/transcriptional regulation/transcription factor (C2H2-ZF)	C2H2 zinc finger transcription factor (original description: none)	4.364
TRIDC5BG082230	15.5.2.1	RNA biosynthesis/transcriptional regulation/MYB transcription factor superfamily/transcription factor (MYB)	transcription factor (MYB) (original description: none)	1.705

TRIDC4AG011220	15.5.2.2	RNA biosynthesis/transcriptional regulation/MYB transcription factor superfamily/transcription factor (MYB-related)	transcription factor (MYB-related) (original description: none)	2.954
TRIDC5BG037960	15.5.22	RNA biosynthesis/transcriptional regulation/transcription factor (WRKY)	transcription factor (WRKY) (original description: none)	5.573
TRIDC5AG007720	15.5.32	RNA biosynthesis/transcriptional regulation/transcription factor (bHLH)	transcription factor (bHLH) (original description: none)	1.704
TRIDC5BG031060	15.5.7.1	RNA biosynthesis/transcriptional regulation/AP2/ERF transcription factor superfamily/transcription factor (ERF)	transcription factor (ERF) (original description: none)	1.886
TRIDC3AG046290	17.4.1.3.12	Protein biosynthesis/translation initiation/Pre-Initiation Complex (PIC) module/eIF3 mRNA-to-PIC binding complex/component eIF3l	component eIF3l of eIF3 mRNA-to-PIC binding complex (original description: none)	7.197
TRIDC6AG005710	18.10.1.4	Protein modification/targeting peptide maturation/endomembrane system/signal peptidase (SPP)	nitrate transporter (NRT2) (original description: none)	18.658
TRIDC6AG034550	18.4.1.12	Protein modification/phosphorylation /TKL protein kinase superfamily/protein kinase (LRR-XII)	protein kinase (LRR-XII) (original description: none)	2.796
TRIDC1AG047410	18.4.1.12	Protein modification/phosphorylation /TKL protein kinase superfamily/protein kinase (LRR-XII)	protein kinase (LRR-XII) (original description: none)	7.092

TRIDC6BG023580	18.4.1.19	Protein modification/phosphorylation/TKL protein kinase superfamily/protein kinase (L-lectin)	Probable LRR receptor-like serine/threonine-protein kinase At4g20450 OS=Arabidopsis thaliana (sp|c0lgq7|y4245_arath : 346.0) & Enzyme classification.EC_2 transferases.EC_2.7 transferase transferring phosphorus-containing group(50.2.7 : 144.3) (original description: none)	1.977
TRIDC7AG012250	18.4.1.24.1	Protein modification/phosphorylation/TKL protein kinase superfamily/G-Lectin protein kinase families/protein kinase (SD-1)	Acid phosphatase 1 OS=Solanum lycopersicum (sp|p27061|ppa1_sollc : 94.0)	1.936
TRIDC5BG010450	18.4.1.25	Protein modification/phosphorylation/TKL protein kinase superfamily/protein kinase (WAK/WAKL)	protein kinase (WAK/WAKL) (original description: none)	6.407
TRIDC5BG010470	18.4.1.25	Protein modification/phosphorylation/TKL protein kinase superfamily/protein kinase (WAK/WAKL)	protein kinase (WAK/WAKL) (original description: none)	7.548
TRIDC3AG040710	18.4.1.30	Protein modification/phosphorylation/TKL protein kinase superfamily/protein kinase (MAP3K-RAF)	protein kinase (MAP3K-RAF) (original description: none)	3.977
TRIDC5BG001040	18.4.1.46	Protein modification/phosphorylation/TKL protein kinase superfamily/protein kinase (DLSV)	protein kinase (DLSV) (original description: none)	6.467
TRIDC3BG013490	18.4.24.1.1	Protein modification/phosphorylation/serine/threonine protein phosphatase superfamily/PPP Fe-Zn-dependent phosphatase families/PP1-class phosphatase	PP1 phosphatase (original description: none)	2.935

TRIDC5AG037820	18.4.24.2.8	Protein modification/phosphorylation/serine/threonine protein phosphatase superfamily/PPM/PP2C Mn/Mg-dependent phosphatase families/clade H phosphatase	clade H phosphatase (original description: none)	6.885
TRIDC4AG019030	18.4.5.3	Protein modification/phosphorylation/CAMK protein kinase superfamily/SNF1-related protein kinase (SnRK3)	SNF1-related protein kinase (SnRK3) (original description: none)	2.001
TRIDC7BG032770	18.4.7.2	Protein modification/phosphorylation/atypical protein kinase families/protein kinase (RIO)	RIO protein kinases (RIOKs) are a relatively conserved family of enzymes implicated in cell cycle control and ribosomal RNA processing.	1.738
TRIDC2BG032750	18.8.1.4	Protein modification/S-glutathionylation/glutathione S-transferase activities/class tau glutathione S-transferase	class tau glutathione S-transferase (original description: none)	1.513
TRIDC2BG059360	19.4.2.1.2	Protein homeostasis/proteolysis/serine-type peptidase activities/S8-class protease (subtilisin) families/protease (SBT2)	protease (SBT2) (original description: none)	20.112
TRIDC5BG079600	19.4.3.1	Protein homeostasis/proteolysis/aspartic-type peptidase activities/A1-class protease (Pepsin)	pepsin-type protease (original description: none)	6.364
TRIDC5BG006190	20.1.3.10	Cytoskeleton organization/microtubular network/Kinesin microtubule-based motor protein activities/motor protein (Kinesin-12)	motor protein (Kinesin-12) (original description: none)	-1.397

TRIDC2AG057350	20.1.5.1.3	Cytoskeleton organization/microtubular network/microtubule dynamics/microtubule (MT) plus-end-tracking/MT plus-end-tracking protein (SPR1)	MT plus-end-tracking protein (SPR1) (original description: none)	2.410
TRIDC2BG010910	21.2.2.1.3.1	Cell wall organization/hemicellulose/xylan/biosynthesis/xylosyltransferase activities/xylosyltransferase (IRX9)	xylosyltransferase (IRX9) (original description: none)	-3.653
TRIDC2BG031640	21.2.4.1	Cell wall organization/hemicellulose/mixed-linked glucan/D-glucan synthase (CSLF)	D-glucan synthase (CSLF) (original description: none)	-1.489
TRIDC2BG042360	21.8.1.2	Cell wall organization/sporopollenin/biosynthesis/acyl-CoA synthetase (ACS5)	acyl-CoA synthetase (ACS5) (original description: none)	20.670
TRIDC6BG021650	22.6.3.1	Vesicle trafficking/target membrane tethering/Exocyst complex/component EXO70	Exo70 is a direct substrate of the extracellular signal-regulated kinases 1/2 (ERK1/2)	-1.426
TRIDC2BG087400	22.6.3.1	Vesicle trafficking/target membrane tethering/Exocyst complex/component EXO70	component EXO70 of Exocyst complex (original description: none)	1.575
TRIDC5AG068850	24.2.1.5	Solute transport/carrier-mediated transport/DMT superfamily/solute transporter (UmamiT)	solute transporter (UmamiT) (original description: none)	-2.528
TRIDC1BG043540	24.2.1.5	Solute transport/carrier-mediated transport/DMT superfamily/solute transporter (UmamiT)	solute transporter (UmamiT) (original description: none)	1.722
TRIDC2AG055510	24.2.2.1.3	Solute transport/carrier-mediated transport/MFS superfamily/SP family/inositol transporter (INT)	inositol transporter (INT) (original description: none)	4.746
TRIDC5AG029700	24.2.2.1.6	Solute transport/carrier-mediated transport/MFS superfamily/SP family/monosaccharide transporter (STP)	monosaccharide transporter (STP) (original description: none)	1.410

TRIDC6AG003780	24.2.2.10	Solute transport/carrier-mediated transport/MFS superfamily/nitrate transporter (NRT2)	nitrate transporter (NRT2) (original description: none)	3.582
TRIDC1AG040150	24.2.2.9	Solute transport/carrier-mediated transport/MFS superfamily/anion transporter (NRT1/PTR)	anion transporter (NRT1/PTR) (original description: none)	1.668
TRIDC4BG056890	24.2.2.9	Solute transport/carrier-mediated transport/MFS superfamily/anion transporter (NRT1/PTR)	anion transporter (NRT1/PTR) (original description: none)	1.754
TRIDC2AG080650	24.2.2.9	Solute transport/carrier-mediated transport/MFS superfamily/anion transporter (NRT1/PTR)	anion transporter (NRT1/PTR) (original description: none)	3.516
TRIDC7BG058370	24.2.3.5.1	Solute transport/carrier-mediated transport/APC superfamily/APC family/amino acid transporter (LAT)	Peroxidase 3 OS=Arabidopsis thaliana (sp|o23044|per3_arath : 380.0)	1.733
TRIDC7BG021730	24.2.4.1.1	Solute transport/carrier-mediated transport/MOP superfamily/MATE family/metabolite transporter (DTX)	DTX/MATE-type Transporter Facilitates Abscisic Acid Efflux	-1.312
TRIDC6AG056910	24.2.6.1	Solute transport/carrier-mediated transport/TOC superfamily/sugar efflux transporter (SWEET)	Peroxidase 2 OS=Zea mays (sp|q9feq8|per2_maize : 211.0)	1.522
TRIDC2AG036110	24.3.9	Solute transport/channels/ligand-gated cation channel (GLR)	ligand-gated cation channel (GLR) (original description: none)	1.406
TRIDC5BG036930	24.3.9	Solute transport/channels/ligand-gated cation channel (GLR)	ligand-gated cation channel (GLR) (original description: none)	1.918
TRIDC1BG044360	26.6.1.1.1	External stimuli response/salinity/SOS (Salt Overly Sensitive) signalling pathway/SOS3-SOS2 signalling/calcium-dependent SOS2 activator (SOS3)	calcium-dependent SOS2 activator SOS3 (original description: none)	-2.432

TRIDC5BG051120	27.1.4	Multi-process regulation/circadian clock system/time-of-day-dependent expressed repressor (PRR)	PRR circadian clock time-of-day-dependent expressed repressor (original description: none)	2.157
TRIDC7AG013410	27.8.1	Multi-process regulation/PEB protein-dependent signalling/regulatory protein (PEBP)	protein kinase (SD-1) (original description: none)	7.519
TRIDC4AG065770	35.1	not assigned/annotated	Disease resistance protein RPM1 OS=Arabidopsis thaliana (sp|q39214|rpm1_arath : 400.0)	-5.008
TRIDC7BG010170	35.1	not assigned/annotated	transcription factor (MADS/AGL) (original description: none)	-3.475
TRIDC6BG012240	35.1	not assigned/annotated	Probable LRR receptor-like serine/threonine-protein kinase At3g47570 OS=Arabidopsis thaliana (sp|c0lgp4|y3475_arath : 238.0)	-2.280
TRIDC6AG011890	35.1	not assigned/annotated	Pentatricopeptide repeat-containing protein At4g02750 OS=Arabidopsis thaliana (sp|q9sy02|pp301_arath : 444.0)	-2.103
TRIDC2BG004420	35.1	not assigned/annotated	Chalcone synthase 2 OS=Secale cereale (sp|p53415|chs2_secce : 132.0)	-2.060
TRIDC7AG072970	35.1	not assigned/annotated	Ubiquitin-40S ribosomal protein S27a-3 OS=Arabidopsis thaliana (sp|p59233|r27ac_arath : 142.0)	-2.033
TRIDC4BG005240	35.1	not assigned/annotated	Uncharacterized protein At4g38062 OS=Arabidopsis thaliana (sp|p0cb23|y4862_arath : 192.0)	-1.921

TRIDC7AG020140	35.1	not assigned/annotated	Probable apyrase 3 OS=Oryza sativa subsp. japonica (sp|q2qye1|apy3_orysj : 591.0) & Enzyme classification.EC_3 hydrolases.EC_3.6 hydrolase acting on acid anhydride(50.3.6 : 113.0) (original description: none)	-1.908
TRIDC6BG054630	35.1	not assigned/annotated	Probable RNA-dependent RNA polymerase 1 OS=Oryza sativa subsp. japonica (sp|q0dxs3|rdr1_orysj : 1255.0)	-1.789
TRIDC1AG039360	35.1	not assigned/annotated	Zinc finger CCCH domain-containing protein 44 OS=Arabidopsis thaliana (sp|q9sd34|c3h44_arath : 216.0)	-1.776
TRIDC6BG017260	35.1	not assigned/annotated	S-(+)-linalool synthase, chloroplastic OS=Oryza sativa subsp. japonica (sp|q6zh94|lins_orysj : 114.0) & Enzyme classification.EC_4 lyases.EC_4.2 carbon-oxygen lyase(50.4.2 : 32.8) (original description: none)	-1.757
TRIDC7BG064160	35.1	not assigned/annotated	Probable E3 ubiquitin-protein ligase LOG2 OS=Arabidopsis thaliana (sp|q9s752|lofg2_arath : 250.0)	-1.581
TRIDC7AG006240	35.1	not assigned/annotated	7-deoxyloganetin glucosyltransferase OS=Gardenia jasminoides (sp|f8wkw1|ugt2_garja : 579.0) & Enzyme classification.EC_2 transferases.EC_2.4 glycosyltransferase(50.2.4 : 232.2) (original description: none)	-1.541

TRIDC7AG075460	35.1	not assigned/annotated	Probable O-methyltransferase 2 OS=Sorghum bicolor (sp|a8qw51|omt2_sorbi : 471.0) & Enzyme classification.EC_2 transferases.EC_2.1 transferase transferring one-carbon group(50.2.1 : 124.1) (original description: none)	-1.511
TRIDC7BG056330	35.1	not assigned/annotated	histone (H2A) (original description: none)	-1.507
TRIDC7BG026890	35.1	not assigned/annotated	metabolite transporter (DTX) (original description: none)	-1.503
TRIDC3BG024680	35.1	not assigned/annotated	Glycerol-3-phosphate acyltransferase 1 OS=Arabidopsis thaliana (sp|q9shj5|gpat1_arath : 214.0)	-1.481
TRIDC7BG068600	35.1	not assigned/annotated	Probable histidine kinase 1 OS=Oryza sativa subsp. indica (sp|a2yfr6|ohk1_orysi : 1377.0)	-1.402
TRIDC1BG021020	35.1	not assigned/annotated	MAR-binding filament-like protein 1-1 OS=Nicotiana tabacum (sp|q9m7j4|mfp1_tobac : 201.0)	-1.351
TRIDC7BG032720	35.1	not assigned/annotated	Thaumatin-like protein 1 OS=Arabidopsis thaliana (sp|a0a1p8b554|thlp1_arath : 134.0)	-1.347
TRIDC4AG012300	35.1	not assigned/annotated	Alpha-dioxygenase 1 OS=Arabidopsis thaliana (sp|q9sgh6|dox1_arath : 276.0)	-1.339
TRIDC2AG066940	35.1	not assigned/annotated	Putative disease resistance protein RGA3 OS=Solanum bulbocastanum (sp|q7xa40|rga3_solbu : 265.0)	-1.322
TRIDC7BG060470	35.1	not assigned/annotated	amino acid transporter (LAT) (original description: none)	-1.322

TRIDC4BG040200	35.1	not assigned/annotated	Small GTPase LIP1 OS=Arabidopsis thaliana (sp|q9c5j9|liip1_arath : 211.0)	-1.300
TRIDC1BG040170	35.1	not assigned/annotated	Probable LRR receptor-like serine/threonine-protein kinase At3g47570 OS=Arabidopsis thaliana (sp|c0lgp4|y3475_arath : 175.0)	1.321
TRIDC6BG067850	35.1	not assigned/annotated	GDSL esterase/lipase At1g71691 OS=Arabidopsis thaliana (sp|q9sf78|gdl29_arath : 166.0)	1.332
TRIDC1BG032670	35.1	not assigned/annotated	Glutathione S-transferase U18 OS=Arabidopsis thaliana (sp|q9fus9|gstui_arath : 111.0)	1.338
TRIDC2AG013320	35.1	not assigned/annotated	Putative disease resistance RPP13-like protein 1 OS=Arabidopsis thaliana (sp|q9lrr4|r13l1_arath : 232.0)	1.350
TRIDC6AG039210	35.1	not assigned/annotated	protein kinase (LRR-XII) (original description: none)	1.399
TRIDC6AG058030	35.1	not assigned/annotated	sugar efflux transporter (SWEET) (original description: none)	1.407
TRIDC2BG046370	35.1	not assigned/annotated	Protein SET DOMAIN GROUP 41 OS=Arabidopsis thaliana (sp|q3ecy6|sdg41_arath : 247.0)	1.414
TRIDC5AG046840	35.1	not assigned/annotated	Rhodanese-like domain-containing protein 14, chloroplastic OS=Arabidopsis thaliana (sp|q94a65|str14_arath : 258.0)	1.436
TRIDC1AG037370	35.1	not assigned/annotated	CASP-like protein 1D1 OS=Oryza sativa subsp. japonica (sp|q6yt98|cspl7_orysj : 95.9)	1.443

TRIDC4BG008640	35.1	not assigned/annotated	Phenolic glucoside malonyltransferase 2 OS=Arabidopsis thaliana (sp|q9lrq8|pmat2_arath : 119.0)	1.470
TRIDC3AG055380	35.1	not assigned/annotated	Receptor protein-tyrosine kinase CEPR2 OS=Arabidopsis thaliana (sp|q9c7t7|cepr2_arath : 589.0)	1.477
TRIDC2AG043690	35.1	not assigned/annotated	Protein SET DOMAIN GROUP 41 OS=Arabidopsis thaliana (sp|q3ecy6|sdg41_arath : 80.9)	1.477
TRIDC2AG041570	35.1	not assigned/annotated	Heavy metal-associated isoprenylated plant protein 22 OS=Arabidopsis thaliana (sp|q93vp2|hip22_arath : 171.0)	1.491
TRIDC3AG006590	35.1	not assigned/annotated	GDSL esterase/lipase At2g27360 OS=Arabidopsis thaliana (sp|q9zqi3|gdl40_arath : 184.0)	1.513
TRIDC1BG025800	35.1	not assigned/annotated	E3 ubiquitin-protein ligase RZFP34 OS=Arabidopsis thaliana (sp|q9ffb6|zfp34_arath : 158.0)	1.522
TRIDC1BG007600	35.1	not assigned/annotated	Protein trichome birefringence-like 11 OS=Arabidopsis thaliana (sp|q5bpj0|tbl11_arath : 260.0)	1.529
TRIDC5AG014710	35.1	not assigned/annotated	L-gulonolactone oxidase 2 OS=Arabidopsis thaliana (sp|q6nq66|gglo2_arath : 562.0)	1.541
TRIDC5AG005640	35.1	not assigned/annotated	Putative disease resistance protein RGA3 OS=Solanum bulbocastanum (sp|q7xa40|rga3_solbu : 248.0)	1.618

TRIDC6AG0 10840	35.1	not assigned/annotated	signal peptidase (SPP) (original description: none)	1.644
TRIDC6BG0 43940	35.1	not assigned/annotated	Probable WRKY transcription factor 19 OS=Arabidopsis thaliana (sp|q9sz67|wrk19_arath : 255.0)	1.715
TRIDC3AG0 22790	35.1	not assigned/annotated	Pathogen-related protein OS=Hordeum vulgare (sp|p16273|prpx_horvu : 345.0)	1.785
TRIDC2BG0 82330	35.1	not assigned/annotated	Heavy metal-associated isoprenylated plant protein 39 OS=Arabidopsis thaliana (sp|o03982|hip39_arath : 83.2)	1.877
TRIDC4AG0 38540	35.1	not assigned/annotated	Dormancy-associated protein 1 OS=Pisum sativum (sp|o22611|drm1_pea : 92.8)	2.006
TRIDC2BG0 15880	35.1	not assigned/annotated	Peroxidase 2 OS=Oryza sativa subsp. indica (sp|a2ypx3|per2_orysi : 358.0)	2.039
TRIDC3BG0 60650	35.1	not assigned/annotated	UDP-glycosyltransferase 91A1 OS=Arabidopsis thaliana (sp|q940v3|u91a1_arath : 80.1)	2.259
TRIDC6BG0 48590	35.1	not assigned/annotated	transcription factor (MADS/AGL) (original description: none)	2.270
TRIDC4BG0 60190	35.1	not assigned/annotated	L-gulonolactone oxidase 2 OS=Arabidopsis thaliana (sp|q6nq66|gglo2_arath : 533.0)	2.584
TRIDC7AG0 37590	35.1	not assigned/annotated	65-kDa microtubule-associated protein 8 OS=Arabidopsis thaliana (sp|q9c7g0|ma658_arath : 397.0)	2.658

TRIDC2AG077130	35.1	not assigned/annotated	Chaperone protein dnaJ C76, chloroplastic OS=Arabidopsis thaliana (sp|q9fmx6|djc76_arath : 271.0)	2.826
TRIDC4AG008190	35.1	not assigned/annotated	MADS-box transcription factor 1 OS=Oryza sativa subsp. indica (sp|a2xdy1|mads1_orysi : 186.0)	2.884
TRIDC7BG019110	35.1	not assigned/annotated	Pathogenesis-related protein 1 OS=Asparagus officinalis (sp|q05736|pr1_aspof : 91.3)	3.286
TRIDC2BG051790	35.1	not assigned/annotated	Probable aldo-keto reductase 2 OS=Oryza sativa subsp. indica (sp|a2xrz0|akr2_orysi : 139.0)	3.467
TRIDC7BG036280	35.1	not assigned/annotated	transcription factor (MADS/AGL) (original description: none)	3.536
TRIDC5AG020540	35.1	not assigned/annotated	Beta-galactosidase 4 OS=Oryza sativa subsp. japonica (sp|q6z6k4|bgal4_orysj : 84.7)	3.690
TRIDC7AG062880	35.1	not assigned/annotated	G-type lectin S-receptor-like serine/threonine-protein kinase At2g19130 OS=Arabidopsis thaliana (sp|o64477|y2913_arath : 587.0) & Enzyme classification.EC_2 transferases.EC_2.7 transferase transferring phosphorus-containing group(50.2.7 : 100.4) (original description: none)	3.786
TRIDC6BG052920	35.1	not assigned/annotated	9-cis-epoxycarotenoid dioxygenase NCED1, chloroplastic OS=Oryza sativa subsp. japonica (sp|q6yvj0|nced1_orysj : 202.0)	3.946

TRIDC6BG011930	35.1	not assigned/annotated	Peroxidase 70 OS=Zea mays (sp|a5h452|per70_maize : 367.0)	4.093
TRIDC5AG047410	35.1	not assigned/annotated	Two-component response regulator-like PRR95 OS=Oryza sativa subsp. japonica (sp|q689g6|prr95_orysj : 374.0)	4.352
TRIDC6BG073190	35.1	not assigned/annotated	Putative disease resistance protein RGA4 OS=Solanum bulbocastanum (sp|q7xa39|rga4_solbu : 201.0)	4.942
TRIDC7BG002180	35.1	not assigned/annotated	Coatomer subunit beta	4.966
TRIDC1BG042910	35.1	not assigned/annotated	Putative disease resistance protein RGA1 OS=Solanum bulbocastanum (sp|q7xa42|rga1_solbu : 137.0)	5.678
TRIDC2BG029870	35.1	not assigned/annotated	Fatty-acid-binding protein 1 OS=Arabidopsis thaliana (sp|q9m1x2|fap1_arath : 155.0)	6.126
TRIDC7AG021890	35.1	not assigned/annotated	RbcX assembly factor involved in RuBisCo assembly (original description: none)	6.328
TRIDC6AG042450	35.1	not assigned/annotated	9-cis-epoxycarotenoid dioxygenase NCED1, chloroplastic OS=Oryza sativa subsp. japonica (sp|q6yvj0|nced1_orysj : 531.0) & Enzyme classification.EC_1 oxidoreductases.EC_1.13 oxidoreductase acting on single donor with incorporation of molecular oxygen (oxygenase)(50.1.12 : 266.4) (original description: none)	6.454

TRIDC7AG0 16880	35.1	not assigned/annotated	PEB protein-dependent signalling regulatory protein (PEBP) (original description: none)	6.708
TRIDC6AG0 21100	35.1	not assigned/annotated	NAC transcription factor NAM-A1 OS=Triticum turgidum subsp. durum (sp|a0spj3|nama1_tritd : 327.0)	6.762
TRIDC6BG0 26600	35.1	not assigned/annotated	Enzyme classification.EC_1 oxidoreductases.EC_1.14 oxidoreductase acting on paired donor with incorporation or reduction of molecular oxygen(50.1.13 : 224.7) & Cytochrome P450 CYP71D313 OS=Panax ginseng (sp|h2dh20|c7d13_pangi : 207.0) (original description: none)	6.773
TRIDC3BG0 39900	35.1	not assigned/annotated	Probable protein phosphatase 2C 8 OS=Oryza sativa subsp. japonica (sp|q5sn75|p2c08_orysj : 162.0)	6.957
TRIDC2BG0 80020	35.1	not assigned/annotated	BTB/POZ and MATH domain-containing protein 1 OS=Arabidopsis thaliana (sp|q8l765|bpm1_arath : 228.0)	7.458
TRIDC6BG0 72880	35.1	not assigned/annotated	A-type cytokinin ARR response negative regulator (original description: none)	7.903

TRIDC2BG000740	50.1.13	Enzyme classification/EC_1 oxidoreductases/EC_1/14 oxidoreductase acting on paired donor with incorporation or reduction of molecular oxygen	Ent-cassadiene C2-hydroxylase OS=Oryza sativa subsp. japonica (sp|q6yv88|c71z7_orysj : 392.0) & Enzyme classification.EC_1 oxidoreductases.EC_1.14 oxidoreductase acting on paired donor with incorporation or reduction of molecular oxygen(50.1.13 : 308.5) (original description: none)	-1.798
TRIDC5AG001540	50.1.13	Enzyme classification/EC_1 oxidoreductases/EC_1/14 oxidoreductase acting on paired donor with incorporation or reduction of molecular oxygen	3-hydroxyindolin-2-one monooxygenase OS=Zea mays (sp|q43250|c71c1_maize : 821.0) & Enzyme classification.EC_1 oxidoreductases.EC_1.14 oxidoreductase acting on paired donor with incorporation or reduction of molecular oxygen(50.1.13 : 342.1) (original description: none)	-1.328
TRIDC4BG000670	50.1.13	Enzyme classification/EC_1 oxidoreductases/EC_1/14 oxidoreductase acting on paired donor with incorporation or reduction of molecular oxygen	Geraniol 8-hydroxylase OS=Swertia mussotii (sp|d1mi46|c76ba_swemu : 413.0) & Enzyme classification.EC_1 oxidoreductases.EC_1.14 oxidoreductase acting on paired donor with incorporation or reduction of molecular oxygen(50.1.13 : 407.6) (original description: none)	-1.307

TRIDC4BG000550	50.1.13	Enzyme classification/EC_1 oxidoreductases/EC_1/14 oxidoreductase acting on paired donor with incorporation or reduction of molecular oxygen	Noroxomaritidine synthase OS=Narcissus aff. pseudonarcissus MK-2014 (sp|a0a140il90|c96t1_narap : 302.0) & Enzyme classification.EC_1 oxidoreductases.EC_1.14 oxidoreductase acting on paired donor with incorporation or reduction of molecular oxygen(50.1.13 : 94.3) (original description: none)	1.480
TRIDC5AG007500	50.1.13	Enzyme classification/EC_1 oxidoreductases/EC_1/14 oxidoreductase acting on paired donor with incorporation or reduction of molecular oxygen	Flavanone 3-dioxygenase 2 OS=Oryza sativa subsp. japonica (sp|q8w2x5|fl3h2_orysj : 158.0) & Enzyme classification.EC_1 oxidoreductases.EC_1.14 oxidoreductase acting on paired donor with incorporation or reduction of molecular oxygen(50.1.13 : 106.5) (original description: none)	2.854
TRIDC2AG008470	50.1.13	Enzyme classification/EC_1 oxidoreductases/EC_1/14 oxidoreductase acting on paired donor with incorporation or reduction of molecular oxygen	Cytochrome P450 99A2 OS=Oryza sativa subsp. japonica (sp|q7x7x4|c99a2_orysj : 303.0) & Enzyme classification.EC_1 oxidoreductases.EC_1.14 oxidoreductase acting on paired donor with incorporation or reduction of molecular oxygen(50.1.13 : 203.1) (original description: none)	3.316

TRIDC2AG0 65830	50.1.13	Enzyme classification/EC_1 oxidoreductases/EC_1/14 oxidoreductase acting on paired donor with incorporation or reduction of molecular oxygen	indolin-2-one monooxygenase OS=Zea mays (sp|q43255|c71c2_maize : 481.0) & Enzyme classification.EC_1 oxidoreductases.EC_1.14 oxidoreductase acting on paired donor with incorporation or reduction of molecular oxygen(50.1.13 : 352.1) (original description: none)	3.701
TRIDC5BG0 08940	50.1.13	Enzyme classification/EC_1 oxidoreductases/EC_1/14 oxidoreductase acting on paired donor with incorporation or reduction of molecular oxygen	Protein DMR6-LIKE OXYGENASE 1 OS=Arabidopsis thaliana (sp|q9zsa8|dlo1_arath : 194.0) & Enzyme classification.EC_1 oxidoreductases.EC_1.14 oxidoreductase acting on paired donor with incorporation or reduction of molecular oxygen(50.1.13 : 110.5) (original description: none)	3.858
TRIDC6BG0 25800	50.1.13	Enzyme classification/EC_1 oxidoreductases/EC_1/14 oxidoreductase acting on paired donor with incorporation or reduction of molecular oxygen	protein kinase (L-lectin) (original description: none)	4.045
TRIDC2BG0 10230	50.1.13	Enzyme classification/EC_1 oxidoreductases/EC_1/14 oxidoreductase acting on paired donor with incorporation or reduction of molecular oxygen	Cytochrome P450 99A2 OS=Oryza sativa subsp. japonica (sp|q7x7x4|c99a2_orysj : 296.0) & Enzyme classification.EC_1 oxidoreductases.EC_1.14 oxidoreductase acting on paired donor with incorporation or reduction of molecular oxygen(50.1.13 : 104.7) (original description: none)	5.467

TRIDC2AG0 19170	50.1.13	Enzyme classification/EC_1 oxidoreductases/EC_1/14 oxidoreductase acting on paired donor with incorporation or reduction of molecular oxygen	Cytochrome P450 709B2 OS=Arabidopsis thaliana (sp|f4ik45|c70b2_arath : 314.0) & Enzyme classification.EC_1 oxidoreductases.EC_1.14 oxidoreductase acting on paired donor with incorporation or reduction of molecular oxygen(50.1.13 : 108.3) (original description: none)	6.187
TRIDC5BG0 26880	50.1.13	Enzyme classification/EC_1 oxidoreductases/EC_1/14 oxidoreductase acting on paired donor with incorporation or reduction of molecular oxygen	Probable 2-oxoglutarate-dependent dioxygenase ANS OS=Arabidopsis thaliana (sp|o80449|diox4_arath : 338.0) & Enzyme classification.EC_1 oxidoreductases.EC_1.14 oxidoreductase acting on paired donor with incorporation or reduction of molecular oxygen(50.1.13 : 140.6) (original description: none)	7.003
TRIDC7AG0 75040	50.2.1	Enzyme classification/EC_2 transferases/EC_2/1 transferase transferring one-carbon group	BURP domain-containing protein 11 OS=Oryza sativa subsp. japonica (sp|q5z5c9|burpb_orysj : 129.0)	-1.482
TRIDC2BG0 06380	50.2.1	Enzyme classification/EC_2 transferases/EC_2/1 transferase transferring one-carbon group	Enzyme classification.EC_2 transferases.EC_2.1 transferase transferring one-carbon group(50.2.1 : 330.0) & Tricetin 3	-1.399
TRIDC4BG0 08630	50.2.3	Enzyme classification/EC_2 transferases/EC_2/3 acyltransferase	Phenolic glucoside malonyltransferase 2 OS=Arabidopsis thaliana (sp|q9lrq8|pmat2_arath : 169.0) & Enzyme classification.EC_2 transferases.EC_2.3 acyltransferase(50.2.3 : 27.6) (original description: none)	2.179

TRIDC2BG089510	50.2.4	Enzyme classification/EC_2 transferases/EC_2/4 glycosyltransferase	UDP-glycosyltransferase 91D1 OS=Stevia rebaudiana (sp|q6vaa8|u91d1_stere : 133.0) & Enzyme classification.EC_2 transferases.EC_2.4 glycosyltransferase(50.2.4 : 103.2) (original description: none)	2.784
TRIDC5BG026280	50.2.4	Enzyme classification/EC_2 transferases/EC_2/4 glycosyltransferase	UDP-glucosyltransferase UGT13248 OS=Hordeum vulgare subsp. vulgare (sp|m0y4p1|ugt13_horvv : 340.0) & Enzyme classification.EC_2 transferases.EC_2.4 glycosyltransferase(50.2.4 : 326.5) (original description: none)	3.572
TRIDC2AG001630	50.2.7	Enzyme classification/EC_2 transferases/EC_2/7 transferase transferring phosphorus-containing group	L-type lectin-domain containing receptor kinase IX.1 OS=Arabidopsis thaliana (sp|q9lxa5|lrk91_arath : 259.0) & Enzyme classification.EC_2 transferases.EC_2.7 transferase transferring phosphorus-containing group(50.2.7 : 101.2) (original description: none)	-1.397
TRIDC6AG000240	50.2.7	Enzyme classification/EC_2 transferases/EC_2/7 transferase transferring phosphorus-containing group	Protein SUPPRESSOR OF NPR1-1 CONSTITUTIVE 4 OS=Arabidopsis thaliana (sp|d7sfh9|lrl26_arath : 371.0) & Enzyme classification.EC_2 transferases.EC_2.7 transferase transferring phosphorus-containing group(50.2.7 : 125.5) (original description: none)	1.610

TRIDC6BG021850	50.2.7	Enzyme classification/EC_2 transferases/EC_2/7 transferase transferring phosphorus-containing group	component EXO70 of Exocyst complex (original description: none)	2.265
TRIDC2AG080440	50.2.7	Enzyme classification/EC_2 transferases/EC_2/7 transferase transferring phosphorus-containing group	Receptor kinase-like protein Xa21 OS=Oryza sativa subsp. indica (sp|q1mx30|xa21_orysi : 412.0) & Enzyme classification.EC_2 transferases.EC_2.7 transferase transferring phosphorus-containing group(50.2.7 : 102.1) (original description: none)	7.432
TRIDC7AG060170	50.2.7	Enzyme classification/EC_2 transferases/EC_2/7 transferase transferring phosphorus-containing group	Enzyme classification.EC_2 transferases.EC_2.8 transferase transferring sulfur-containing group(50.2.8 : 233.1) & Cytosolic sulfotransferase 15 OS=Arabidopsis thaliana (sp|q8l5a7|sot15_arath : 169.0) (original description: none)	7.578
TRIDC2BG034440	50.2.7	Enzyme classification/EC_2 transferases/EC_2/7 transferase transferring phosphorus-containing group	Enzyme classification.EC_2 transferases.EC_2.7 transferase transferring phosphorus-containing group(50.2.7 : 23.9) (original description: none)	7.837
TRIDC7AG059380	50.2.8	Enzyme classification/EC_2 transferases/EC_2/8 transferase transferring sulfur-containing group	cytosolic polyamine oxidase (PAO1) (original description: none)	-2.280

TRIDC1AG037530	50.2.8	Enzyme classification/EC_2 transferases/EC_2/8 transferase transferring sulfur-containing group	Enzyme classification.EC_2 transferases.EC_2.8 transferase transferring sulfur-containing group(50.2.8 : 211.0) & Cytosolic sulfotransferase 1 OS=Arabidopsis thaliana (sp|q9fg94|sot1_arath : 149.0) (original description: none)	-1.782
TRIDC5BG011000	50.3.1	Enzyme classification/EC_3 hydrolases/EC_3/1 hydrolase acting on ester bond	Fructose-1,6-bisphosphatase, cytosolic OS=Saccharum hybrid (sp|q43139|f16p2_sachy : 172.0) & Enzyme classification.EC_3 hydrolases.EC_3.1 hydrolase acting on ester bond(50.3.1 : 78.7) (original description: none)	6.045
TRIDC2AG078730	50.3.2	Enzyme classification/EC_3 hydrolases/EC_3/2 glycosylase	4-hydroxy-7-methoxy-3-oxo-3,4-dihydro-2H-1,4-benzoxazin-2-yl glucoside beta-D-glucosidase 1d, chloroplastic OS=Triticum aestivum (sp|d5mtf8|hgl1d_wheat : 405.0) & Enzyme classification.EC_3 hydrolases.EC_3.2 glycosylase(50.3.2 : 111.2) (original description: none)	-1.360
TRIDC1AG061460	50.3.2	Enzyme classification/EC_3 hydrolases/EC_3/2 glycosylase	Polygalacturonase At1g48100 OS=Arabidopsis thaliana (sp|q949z1|pglr4_arath : 112.0) & Enzyme classification.EC_3 hydrolases.EC_3.2 glycosylase(50.3.2 : 64.6) (original description: none)	7.408

TRIDC7AG019910	50.3.6	Enzyme classification/EC_3 hydrolases/EC_3/6 hydrolase acting on acid anhydride	S-norcoclaurine synthase 1 OS=Coptis japonica (sp|a2a1a0|ncs1_copja : 103.0)	1.833
TRIDC6BG012300	50.4.2	Enzyme classification/EC_4 lyases/EC_4/2 carbon-oxygen lyase	Pentatricopeptide repeat-containing protein At1g02060, chloroplastic OS=Arabidopsis thaliana (sp|o81908|ppr2_arath : 401.0)	6.570

Table S4. Gene ontology enrichment analysis by R package GOSeq, significantly enriched (FDR ≤ 0.05)

Ontology	GO Term	Description	Overrepresentation FDR	Overrepresentation (-Log FDR)	Genes in category	Total genes in the category
MF	GO:0020037	heme binding	8.13E-04	3.090	8	1407
CC	GO:0010287	plastoglobule	9.70E-04	3.013	2	29
MF	GO:0031956	medium-chain fatty acid-CoA ligase activity	2.97E-03	2.528	1	2
BP	GO:0046949	fatty-acyl-CoA biosynthetic process	2.97E-03	2.528	1	2
MF	GO:0008429	phosphatidylethanolamine binding	6.30E-03	2.201	1	4
MF	GO:0004467	long-chain fatty acid-CoA ligase activity	6.55E-03	2.184	1	4
MF	GO:0016702	oxidoreductase activity, acting on single donors with incorporation of molecular oxygen, incorporation of two atoms of oxygen	7.29E-03	2.137	2	77
BP	GO:0042744	hydrogen peroxide catabolic process	7.37E-03	2.133	3	291
CC	GO:0098807	chloroplast thylakoid membrane protein complex	8.18E-03	2.087	1	6
BP	GO:0055114	oxidation-reduction process	9.52E-03	2.021	11	3324
BP	GO:0010229	inflorescence development	1.22E-02	1.914	1	7
BP	GO:0080110	sporopollenin biosynthetic process	1.41E-02	1.852	1	10

BP	GO:0048573	photoperiodism, flowering	1.44E-02	1.842	1	10
BP	GO:0071482	cellular response to light stimulus	1.49E-02	1.828	1	10
MF	GO:0016872	intramolecular lyase activity	1.66E-02	1.780	1	12
BP	GO:0000160	phosphorelay signal transduction system	1.72E-02	1.765	2	133
MF	GO:0004601	peroxidase activity	2.18E-02	1.663	3	434
CC	GO:0009507	chloroplast	2.19E-02	1.659	5	1012
BP	GO:0048510	regulation of timing of transition from vegetative to reproductive phase	2.50E-02	1.602	1	16
BP	GO:0006979	response to oxidative stress	3.04E-02	1.518	3	493
CC	GO:0009524	phragmoplast	3.09E-02	1.510	1	19
MF	GO:0004834	tryptophan synthase activity	3.17E-02	1.499	1	21
MF	GO:0005504	fatty acid binding	3.54E-02	1.450	1	30
CC	GO:0005819	spindle	3.64E-02	1.439	1	21
MF	GO:0016705	oxidoreductase activity, acting on paired donors, with incorporation or reduction of molecular oxygen	3.95E-02	1.403	4	877
BP	GO:0006568	tryptophan metabolic process	4.12E-02	1.385	1	29
CC	GO:0009941	chloroplast envelope	4.92E-02	1.308	2	228
